# Sphaerococcenol A Derivatives: Design, Synthesis, and Cytotoxicity

**DOI:** 10.3390/md22090408

**Published:** 2024-09-05

**Authors:** Dídia Sousa, Milene A. G. Fortunato, Joana Silva, Mónica Pingo, Alice Martins, Carlos A. M. Afonso, Rui Pedrosa, Filipa Siopa, Celso Alves

**Affiliations:** 1 MARE—Marine and Environmental Sciences Centre/ARNET—Aquatic Research Network, ESTM, Politécnico de Leiria, 2520-614 Peniche, Portugal; 2 Research Institute for Medicines (iMed.ULisboa), Faculty of Pharmacy, Universidade de Lisboa, Av. Prof. Gama Pinto, 1649-003 Lisboa, Portugal

**Keywords:** marine natural products, anticancer, apoptosis, reactive oxygen species, algae, hemi-synthesis

## Abstract

Sphaerococcenol A is a cytotoxic bromoditerpene biosynthesized by the red alga *Sphaerococcus coronopifolius*. A series of its analogues (**1**–**6**) was designed and semi-synthesized using thiol-Michael additions and enone reduction, and the structures of these analogues were characterized by spectroscopic methods. Cytotoxic analyses (1–100 µM; 24 h) were accomplished on A549, DU-145, and MCF-7 cells. The six novel sphaerococcenol A analogues displayed an IC_50_ range between 14.31 and 70.11 µM on A549, DU-145, and MCF-7 malignant cells. Compound **1**, resulting from the chemical addition of 4-methoxybenzenethiol, exhibited the smallest IC_50_ values on the A549 (18.70 µM) and DU-145 (15.82 µM) cell lines, and compound **3**, resulting from the chemical addition of propanethiol, exhibited the smallest IC_50_ value (14.31 µM) on MCF-7 cells. The highest IC_50_ values were exhibited by compound **4**, suggesting that the chemical addition of benzylthiol led to a loss of cytotoxic activity. The remaining chemical modifications were not able to potentiate the cytotoxicity of the original compounds. Regarding A549 cell viability, analogue **1** exhibited a marked effect on mitochondrial function, which was accompanied by an increase in ROS levels, Caspase-3 activation, and DNA fragmentation and condensation. This study opens new avenues for research by exploring sphaerococcenol A as a scaffold for the synthesis of novel bioactive molecules.

## 1. Introduction

Cancer is one of the deadliest diseases in the world, having a huge impact on global health and representing a critical barrier to the life expectancy increase. The GLOBOCAN predicts the occurrence of 28.4 million new cases by 2040, corresponding to an increase of 47% compared to 2020 [1]. Assuming the current incidence trends, it is expected that this value will duplicate until 2070, tending to be one of the main causes of precocious death (>70 years old) [2]. Distinct therapeutic strategies such as classical therapies, e.g., surgery, radiotherapy, immunotherapy, targeted therapy, and chemotherapy, and more recently advanced approaches such as stem cell therapy, ablation therapy, and nanoparticles, among others, have been used [3,4]. Some of them are based on the induction of apoptosis through different stimuli, including radiation, hypoxia, toxins, hyperthermia, the production of reactive oxygen species, the activation of death receptors, etc., aiming at the effective elimination of malignant cells [5]. Despite the advances in the last decades, drug resistance to classical and/or new anticancer medicines and the side effects that result from non-specific treatments leading to healthy tissue damage continue to be one of the major cornerstones in cancer treatment [6]. Hence, in pursuing an increase in the quality and life expectancy of oncologic patients, it is imperative to persistently search and develop novel therapeutic tools with heightened efficacy and impact in cancer treatments, including new anticancer drugs. 

Between 1981 and 2019, a total of 247 drugs with an indication for cancer treatment were approved, and their origins were traced back to compounds of natural provenance. Among these, 18 were developed directly from unaltered natural compounds’ structures, while 29 were the result of a synthesis. Furthermore, 36 drugs were meticulously designed to mimic natural compounds, and an additional 43 were formulated using derivatives sourced from natural precursors [7]. These data attest the key role of nature as a source of chemical structures with anticancer potential, inspiring the development of innovative therapeutic agents. Opposite to the terrestrial environment, the marine environment remains poorly explored but exhibits a rich biological and chemical diversity, key factors in discovering bioactive compounds with pharmacological potential [8]. Due to their great diversity, marine natural products have shown an immense potential to inspire the development of innovative drugs with distinct mechanisms of action and great effectiveness in the treatment of many diseases [9]. However, their translation from basic scientific findings in the laboratory to clinical trials has been limited by several factors, such as low stability, bioavailability, water solubility, efficacy, pharmacokinetics, etc. [10]. To overcome these restrictions, the synthesis or hemi-synthesis of analogous molecules of resemblant nature with slight structural deviations can enhance the potency and efficacy of the inherent properties exhibited by a given compound, while concurrently mitigating its limitations.

In recent years, algae have been extensively studied as a source of bioactive compounds with health-beneficial properties such as antioxidant, antimicrobial, anti-inflammatory, immunomodulatory, and anticancer activities [11,12]. The red alga *Sphaerococcus coronopifolius*, Stackhouse 1797, has been revealed to be a great source of diterpenes, most of them brominated, with diverse molecular structures [13]. Sphaerococcenol A is one of the major compounds found in this algae, which is characterized to possess a sphaerane carbon skeleton and has revealed distinct biological activities, including anti-malarial [14] and antitumor [15,16,17,18] properties. Regarding cytotoxic activities, sphaerococcenol A has been displayed to affect the cell viability of different malignant cell lines in 2D, 3D, and co-culture models and stimulate the production of H_2_O_2_ and apoptosis [15,16,17,18]. It also exhibited a cytostatic effect on human apoptosis-resistant U373 cells, inhibiting the cell entry into the mitosis phase [18].

**Scheme 1:**
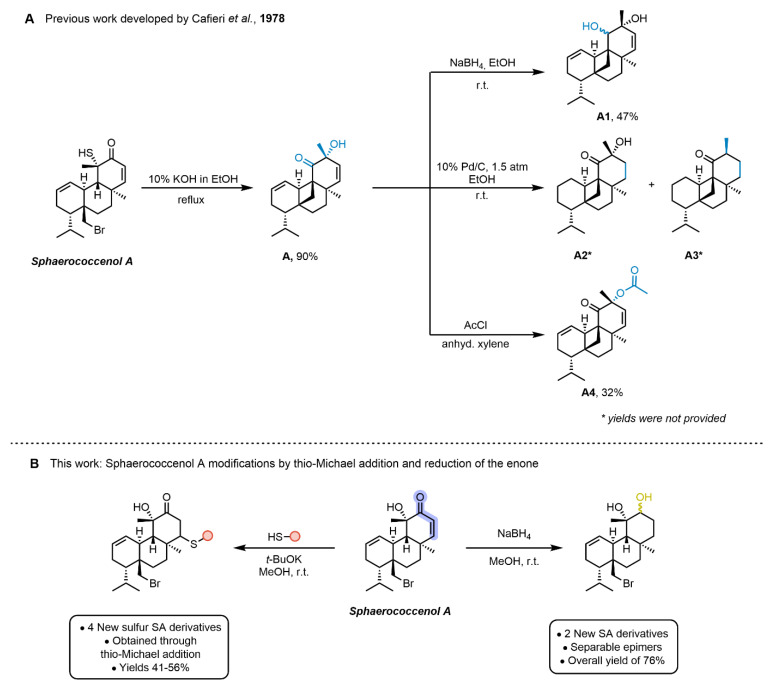
Work on sphaerococcenol A reactions performed by Cafieri and collaborators [19] (**A**). Chemical modifications were performed in this study using sphaerococcenol A as scaffold (**B**).

Despite the interesting activities evidenced by sphaerococcenol A, the hemi-synthesis of new analogues based on its structure was only approached by Cafieri and collaborators in 1978 (Scheme 1A) [19]. A base-catalyzed sphaerococcenol A rearrangement allowed the formation of the new hemi-synthetic compound A, which originated four derivatives (A1–A4) through its reduction, hydrogenation, and acetylation (Scheme 1A). Herein, we report for the first time the synthesis of four novel sulfur-containing sphaerococcenol A analogues (Scheme 1B). Sulfur-containing drugs present an important role in different medicinal areas (e.g., dexlansoprazole, a proton pump inhibitor; emtricitabine, an inhibitor of reverse transcriptase; ceritinib, an antineoplastic kinase inhibitor; and sugammadex, a selective relaxant binding agent) and constitute a large portion of newly approved drugs by the FDA [20]. In addition, sphaerococcenol A derivatives resulting from its reduction were also prepared (Scheme 1B). The cytotoxicity of the novel sphaerococcenol A analogues was further studied in malignant cell lines derived from lung (A549), breast (MCF-7), and prostate (DU-145) tissues as well as biomarkers related to oxidative stress and apoptosis.

## 2. Results

### 2.1. Hemi-Synthesis of New Sphaerococcenol A Analogues

**Scheme 2:**
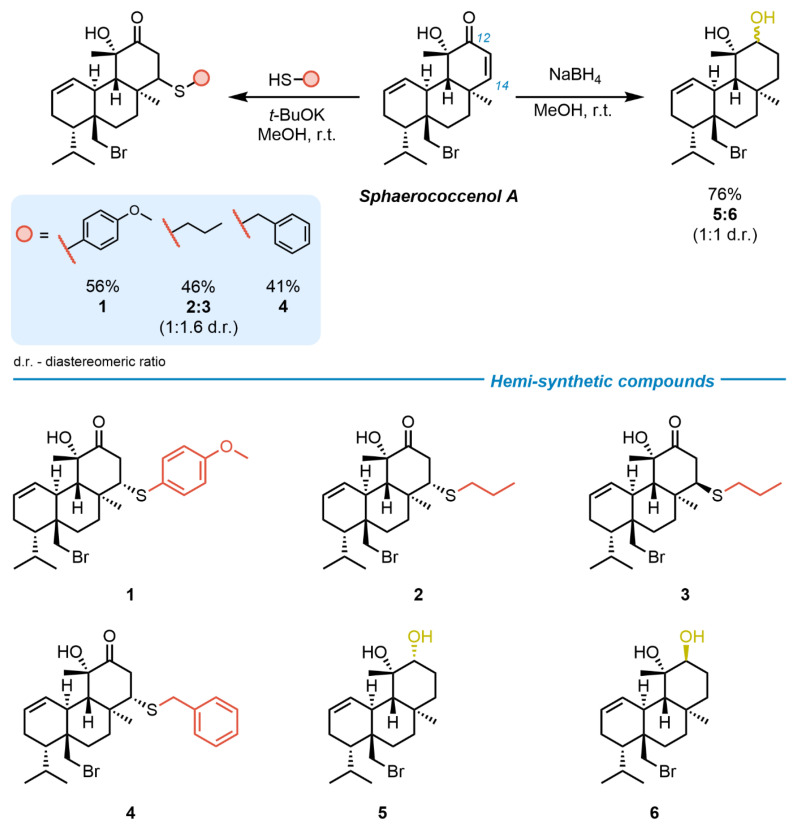
Hemi-synthetic compounds of sphaerococcenol A obtained by thio-Michael addition and reduction.

The synthesis of novel sphaerococcenol A derivatives (**1**–**6**) was achieved by the reaction on the enone function via thiol-Michael addition and reduction (Scheme 2).

**Figure 1:**
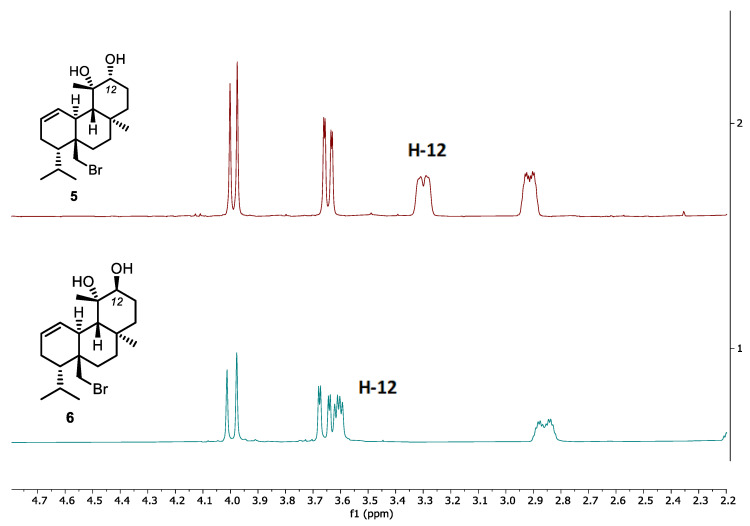
Partial stacking ^1^H NMR spectra of diastereosiomers **5** and **6**.

Thio-Michael addition allowed the preparation of four novel sulfur derivatives of sphaerococcenol A (**1**–**4**) (Scheme 2). The reactions were performed using the corresponding thiol nucleophile in methanol in the presence of potassium *tert*-butoxide (*t*-BuOK). In the case of using 4-methoxybenzenethiol and benzylthiol as the nucleophile, compounds **1** and **4** were obtained at yields of 56% and 41%, respectively. While using propanethiol, a mixture of diastereomers was delivered at a yield of 46% with a diastereomeric ratio of 1:1.55. The sphaerococcenol A reduction with sodium borohydride in methanol also resulted in a mixture of diastereomers with a 1:1 diastereomeric ratio. The new hydroxyl analogues (**5** and **6**) were obtained at a yield of 76% (Scheme 2). The proposed structure of the new hemi-synthetic analogues was assigned based on NMR and HRMS data, and the purity was checked by HPLC and/or ^1^H-NMR and proved to be higher than 85%. The relevant protons for structural identification were H-14 and H-13 for compounds **1**–**4** and H-12 for compounds **5**–**6** (see Supporting Information, Table S1). For diastereoisomers **5** and **6**, a change in the corresponding chemical shifts for H-12 is visible (**5**: δH12 3.30 ppm; **6**: δH12 3.61 ppm, Figure 1). Compound **5** corresponds to the diastereoisomer with the hydroxyl group behind the plane and **6** with the hydroxyl group to the front of the plane.

### 2.2. Cytotoxicity on Malignant Cell Lines

The cytotoxicity of sphaerococcenol A and its derivatives (**1**–**6**) was evaluated in A549, DU-145, and MCF-7 cells. The results are presented in Table 1.

In the malignant cell lines DU-145, MCF-7, and A549, sphaerococcenol A displayed IC_50_ values of 3.75, 6.53, and 14.99 µM, respectively (Table 1). Between the synthesized derivatives (**1**–**6**), compounds **1**, **3**, and **5** exhibited the smallest IC_50_ values across the three cell lines. Amongst the compounds tested, compound **4** and compound **2** showed the highest IC_50_ values. Specifically, compound **4** displayed the highest IC_50_ values in the A549 (35.45 µM) and DU-145 (70.11 µM) cell lines, while compound **2** demonstrated the highest IC_50_ value among the MCF-7 cells (70.26 µM). Regarding the selectivity index, the original compound exhibited the highest value on DU-145 (10.0) and MCF-7 (5.7) malignant cells followed by compound **3** and compound **5** on MCF-7 (4.0) and DU-145 (4.4.) cells, respectively. On the other hand, compound **4** displayed the smallest selectivity index (0.6–1.3). Since analogue **1** demonstrated heightened potency in all three cell lines, with an IC_50_ value that closely paralleled the one obtained from the original compound in the A549 cell line and a selectivity index around 2, further studies were conducted in A549 cells to investigate the potential mechanisms of action underlying the cytotoxic activity previously observed with sphaerococcenol A.

### 2.3. Effects of Analogue 1 on Cell Viability and Mitochondrial Function

**Figure 2:**
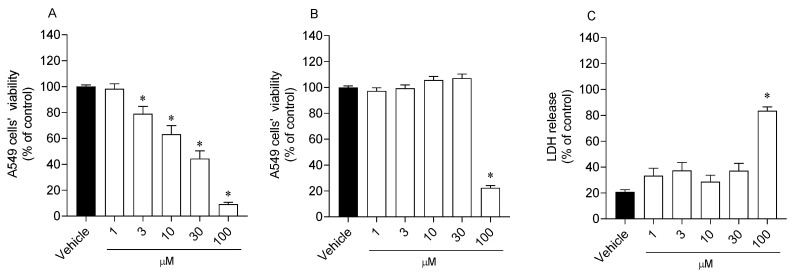
Effects of compound 1 (1–100 µM; 24 h) in A549 cell viability estimated by MTT (**A**), calcein-AM (**B**), and LDH (**C**) assays. Symbols represent significant differences (ANOVA, Dunnett’s test, *p*  <  0.05) when compared to vehicle. At least three independent experiments were carried out in triplicate.

The effects of compound **1** on different cellular targets of A549 cells were estimated by the MTT, LDH, and calcein-AM assays (Figure 2).

The treatment with compound **1** for 24 h induced a significant progressive decline in the mitochondrial function of A549 cells starting at the concentration of 3 µM, with a more pronounced reduction at 100 µM (Figure 2A). On the other hand, the effects of compound **1** revealed by calcein-AM and LDH assays on A549 cell viability were only significant when tested at the maximum concentration (Figure 2B,C).

### 2.4. Reactive Oxygen Species Levels

**Figure 3:**
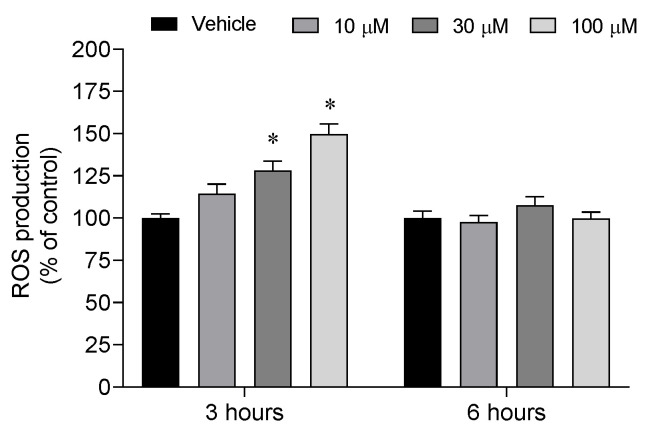
Reactive oxygen species levels produced by A549 cells following treatment with compound 1 (10, 30, and 100 µM) for 3 and 6 h. Symbols represent significant differences (two-way ANOVA, Bonferroni’s test, *p*  <  0.05) when compared to vehicle. At least three independent experiments were carried out in triplicate.

Reactive oxygen species levels were estimated on A549 cells following treatment with compound **1** (10, 30, and 100 µM) for 3 and 6 h (Figure 3).

The A549 cells increased the production of reactive oxygen species after treatment with compound **1** for 3 h when tested at 30 and 100 µM (Figure 3). After a span of 6 h, no significant differences were observed in the levels of ROS when compared with the vehicle.

### 2.5. Mitochondrial Dysfunction and Apoptosis

**Figure 4:**
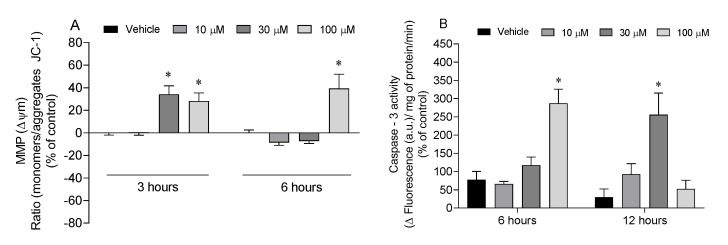

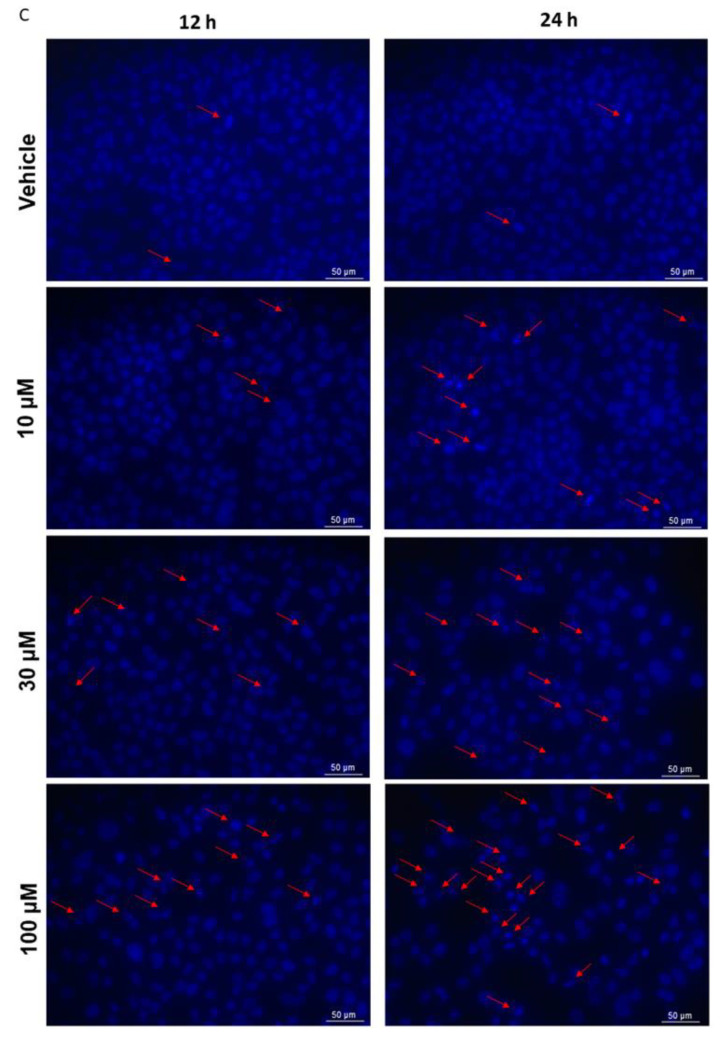
Effects of compound **1** treatment (10, 30, and 100 µM) on A549 cells regarding alterations in mitochondrial membrane potential after 3 and 6 h (**A**), Caspase-3 activity after 6 and 12 h (**B**), and alterations in DNA morphology after 12 and 24 h (**C**). Symbols represent significant differences (two-way ANOVA, Bonferroni’s test, *p*  <  0.05) when compared to vehicle. At least three independent experiments were carried out in triplicate. Images are representative of one well of each treatment condition and were obtained using an inverted fluorescence microscope at ×400. Red arrows indicate alterations in DNA compared to vehicle.

Biomarkers related to mitochondrial dysfunction and apoptosis, including alterations in the mitochondrial membrane potential, Caspase-3 activity, and changes in nuclear morphology, were evaluated in A549 cells following the treatment with compound **1** at distinct times (Figure 4A–C).

Analogue **1** induced a depolarization of the mitochondrial membrane potential of A549 malignant cells after treatment for 3 h at 30 and 100 µM. After 6 h of exposure, only the concentration of 100 µM maintained the effect (Figure 4A). Concerning the activity of Caspase-3, it is possible to observe a stimulation of its activity after 6 h of exposure for a concentration of 100 µM, and likewise after 12 h for a concentration of 30 µM (Figure 4B). All three concentrations induced DNA fragmentation and condensation after 12 and 24 h of treatment, which becomes evident in the regions demarcated by red arrows. The effects were more marked at the concentration of 100 µM (Figure 4C).

## 3. Discussion

Over the centuries, nature has been revealed to be an exceptional reservoir of new and uncommon chemical structures, on which the development of innovative drugs with distinct mechanisms of action was achieved, especially for the treatment of infectious diseases and cancer [21]. Despite the great pharmacological potential evidenced by natural products on laboratory assays, their translation to clinical applications is challenging due distinct factors [22]. However, these chemical structures represent a valuable source of scaffolds on which the development of new analogues can be inspired, improving their efficacy and mitigating limiting factors such as stability, bioavailability, solubility, pharmacokinetics, metabolism, toxicity, etc. [10]. The modification of natural products by semi-synthesis has been revealed to be a starting point for the generation of new analogue libraries with distinct properties from the original compounds, creating new lead structures for drug discovery [23]. Despite the red seaweed *Sphaerococcus coronopifolius* being revealed to be a prolific source of terpenes, most of them halogenated and with cytotoxic properties, there are only two studies that explore its chemical diversity as scaffolds for the synthesis of novel hemi-synthetic compounds [11,19,24]. As previously referenced, sphaerococcenol A was subjected to a base-catalyzed rearrangement, originating the new hemi-synthetic compound A, which by reduction, hydrogenation, and acetylation originated four new A analogues [19]. In addition to this, in 2022, Prousis et al. [24] reported the synthesis of hemi-synthetic analogues of bromosphaerol by reactions such as epoxidation and sequential hydroboration/oxidation. The newly synthesized bromosphaerol derivatives were evaluated regarding their antifouling activity [24]. Herein, we reported for the first time the synthesis of four novel sulfur-containing sphaerococcenol A analogues (**1**–**4**) and two derivatives (**5** and **6**) resulting from its enone reduction, aiming to improve the cytotoxic activity of the original compound. This library of new hemi-synthetic sphaerococcenol A derivatives allowed us to study key structure modifications, namely the effects of losing the enone function, the insertion of an alcohol function group at C-12 and its respective orientation (derivatives **5** and **6**)**,** and the insertion of sulfur-containing moieties at C-14 (derivatives **1**–**4**). In the latter, the aliphatic and aromatic thiol substituent can also be compared.

The thio-Michael addition provided four novel sphaerococcenol A derivatives (**1**–**4**). Using 4-methoxybenzenethiol and benzylthiol as nucleophiles, only one epimer was obtained (**1** and **4**), while using propanethiol, two epimers were provided (**2** and **3**), resulting from the attack on both faces of the enone.

Sphaerococcenol A has a complex structure with distinct functional groups, such as alcohols, enones, olefins, and alkyl halides, which can undergo different chemical transformations. Thiols, in addition to engaging in the thio-Michael addition to enones, can also attack alkyl halides in a S_N_2 fashion. Since sphaerococcenol A has an enone and an alkyl bromide function, the bromine substitution product could be expected. We proved the single formation of the thio-Michael product by subjecting the sphaerococcenol A to a large excess of thiol (20.0 equiv) and just observing the correspondent thio-Michael product. Additionally, when 1-bromo-2,2-dimethylpropane (a neopentyl bromide) was submitted to the standard reaction’s conditions (2.0 equiv of thiol), it did not react. Concerning the enone reduction using sodium borohydride, two alcohol epimers (**5** and **6**) were originated in the same ratio derived from the 1,2- and 1,4-reduction.

The configuration of the newly formed stereocenters, C-14 for compounds **1**–**4** and C-12 for compounds **5** and **6**, was inferred by chemical shift values, an analysis of the coupling constants, and by a comparison with similar compounds isolated from the red alga *Sphaerococcus coronopifolius* [25] (Table S1). Compounds **1**, **2**, and **4** present high coupling constants of H-14 with the vicinal protons H-13α and H-13β. This is compatible with H-14 being in an axial configuration. On the opposite side, compound **3** presents a low coupling constant between H-14 and H-13β, indicative of an equatorial–equatorial configuration. This supported the equatorial configuration of H-14. Compounds **5** and **6** are epimers, and by comparing the coupling constants of H-12 with H-13, it is noticeable that **5** has a higher coupling constant than **6**, and this is consistent with H-12 being on an axial configuration on **5**. These findings are also supported by a comparison to similar compounds isolated from *Sphaerococcus coronopifolius* that have the same stereochemistry (Table S1).

Concerning the cytotoxic activities, the synthesized compounds displayed moderate effects ranging from 14.31 to 70.11 µM, never exceeding the IC_50_ values exhibited by the treatment with sphaerococcenol A. The only exceptions were observed with compounds **2** and **4**, which displayed significant differences when compared with the original compound. The extra methylene group in compound **4** seems to not be essential for sphaerococcenol A cytotoxicity, since compound **1** maintained similar activity to sphaerococcenol A and compound **4** lost the activity. Developing drugs with high selectivity for cancer cells is essential for creating more effective treatments for cancer patients. Achieving this selectivity not only enhances therapeutic efficacy but also minimizes damage to healthy tissues, thereby reducing side effects and improving patient outcomes. In this study, the original compound exhibited the highest selectivity index in DU-145 and MCF-7 cells, with only compound **5** surpassing it in the A549 cell line. The chemical modifications introduced did not appear to enhance the selectivity index. However, evaluating these compounds in additional cellular models could provide a more comprehensive understanding of their cytotoxic potential.

The effects of compounds on cell viability have been estimated through different assays, which have distinct cellular targets [26]. The MTT assay is widely used to estimate cellular metabolism through its reduction by mitochondrial dehydrogenases. On the other hand, the LDH assay allows us to understand if the cell membrane integrity was lost by measuring the levels of lactate dehydrogenase that leak from the cytoplasm into the culture medium [27]. In turn, calcein-AM is a lipid-soluble diester fluorogenic esterase substrate that can be converted by intracellular esterases in a hydrophilic, strongly fluorescent compound that is retained in the cytoplasm staining the viable cells [28]. Here, analogue **1** was able to induce a significant reduction in the cell metabolism at concentrations higher than 3 µM, while the significant effects estimated by the calcein-AM and LHD assays were only significant at the maximum concentration of 100 µM. These data suggest that compound **1** can target the cell metabolism instead to induce a disruption of the cell membrane.

The abnormal levels of ROS are often associated with changes in cellular metabolic activities and the occurrence of oxidative stress conditions, which affects both the development and maintenance of cancer [29]. The stimulation of ROS generation until achieving unsustainable cellular levels has been explored as a therapeutic strategy in cancer, leading to the activation of antitumorigenic signaling pathways and inducing cell senescence and cell death by apoptosis [30]. Usually, malignant cells display higher ROS levels due to their elevated metabolic rate becoming more vulnerable to further ROS damage than normal cells, which can regulate these levels through the antioxidant defense system [31,32,33]. Previous studies carried out with sphaerococcenol A in MCF-7 cells suggested that the decrease in cell viability was accompanied by the production of hydrogen peroxide, changes in mitochondrial membrane potential, the stimulation of Caspase-9 activity, and nuclear morphology alterations [15]. Although compound **1** did not potentiate the cytotoxic effects of the original compound in A549 cells, it seems that it induces cell viability reduction by similar mechanisms of the original compound. It was also able to increase the production of ROS, decrease the mitochondrial membrane potential, stimulate the Caspase-3 activity, and induce DNA fragmentation and condensation. However, to establish a potential relation between the increase in ROS levels mediated by compound **1** and the activation of apoptosis, it will be essential to treat the cells with compound **1** in the presence of an antioxidant molecule such as N-acetylcysteine (NAC) to understand if the activation of apoptosis is directly activated by the ROS production or by other mechanisms. Furthermore, although the new derivatives do not enhance the cytotoxic effects of sphaerococcenol A, other factors such as solubility, stability, and additional biological activities, such as anti-inflammatory, immunomodulatory, and anti-angiogenic properties, should be explored in future studies to assess their potential contributions to cancer treatment.

## 4. Materials and Methods

### 4.1. Hemi-Synthesis of Analogues

#### 4.1.1. Reagents

Reagents and solvents were purchased from commercial sources. All solvents were distilled before use. Methanol was distilled with calcium hydride under nitrogen and stored with 3 Å molecular sieves under inert gas. NMR spectra were recorded in a Bruker Fourier 300 (Bruker, Billerica, MA, USA) or a Bruker 400 (Bruker, Billerica, MA, USA) using CDCl_3_ as a deuterated solvent. All coupling constants are expressed in Hz and chemical shifts (δ) in ppm. Multiplicities are given as s (singlet), d (doublet), dd (double doublet), dt (double triplet), t (triplet), td (triple triplet), tt (triple triplet), q (quartet), quint (quintuplet), and m (multiplet). High-Resolution Mass spectra were recorded in a Thermo Scientific Q Exactive hybrid quadrupole-Orbitrap mass spectrometer (Thermo Scientific Q Exactive Plus, Waltham, MA, USA). Reaction mixtures were analyzed by thin-layer chromatography using Merck silica gel 60F254 aluminum plates and visualized by UV light or stained with phosphomolybdic acid stain. Column chromatography was performed with silica gel Geduran^®^ Si 60 (0.040–0.063 mm) purchased from Merck. Compounds **2** and **3** were purified using the HPLC equipment Thermo Scientific™ UltiMate™ 3000 UHPLC coupled with a semi-preparative column (Surf C18 100A 10 µM 250 × 10).

#### 4.1.2. Extraction and Isolation of Sphaerococcenol A

*Sphaerococcus coronopifolius* specimens were collected from Berlenga Nature Reserve (39°24′47.9″ N 9°30′28.2″ W), Peniche, Portugal, with previous authorization of the Instituto da Conservação da Natureza e das Florestas (ICNF). Freeze-dried and powered biomass (60 g) was sequentially extracted with 1.2 L of a solution of 1:1 dichloromethane–methanol (72 h) and then with 1.2 L of methanol (48 h). The resulting extracts (1.43 g) were purified by silica gel chromatography (eluent: *n*-hexane/ethyl acetate (9:1)), yielding pure sphaerococcenol A (190 mg, 13% wt). The spectral data are in accordance with the literature [34]. ^1^H NMR (300 MHz, CDCl_3_) δ 6.81 (d, *J* = 9.9 Hz, 1H), 6.05 (dd, *J* = 10.0, 4.1 Hz, 2H), 5.76–5.69 (m, 1H), 3.88 (d, *J* = 10.7 Hz, 1H), 3.70 (dd, *J* = 10.7, 2.0 Hz, 1H), 2.99 (s, 1H), 2.88 (ddt, *J* = 12.2, 4.8, 2.4 Hz, 1H), 2.22–2.09 (m, 1H), 2.02–1.87 (m, 3H), 1.85–1.42 (m, 6H), 1.32 (s, 3H), 1.07 (s, 3H), 0.93 (dd, *J* = 14.3, 6.8 Hz, 6H). ^13^C NMR (75 MHz, CDCl_3_) δ 204.1, 162.9, 128.4, 127.0, 124.6, 75.5, 45.7, 42.2, 40.4, 40.0, 37.0, 35.6, 33.2, 31.5, 26.1, 26.0, 24.7, 22.5, 21.5, 19.6.

#### 4.1.3. General Procedure for Thio-Michael Addition to Sphaerococcenol A (GP1)

In a Schlenk equipped with a stir bar, flame-dried and purged under an argon atmosphere, a solution of sphaerococcenol A (10.0 mg, 0.026 mmol) was prepared in distilled methanol (0.9 mL) with the respective thiol (2.0 equiv, 0.052 mmol) and potassium tert-butoxide (1.5 equiv, 0.039 mmol). The mixture was stirred at room temperature (25 °C). After consumption of sphaerococcenol A, a saturated solution of sodium bicarbonate (3 mL) was added to the mixture, and an extraction was performed using dichloromethane (4 × 3 mL). The organic phase was dried with anhydrous magnesium sulphate, filtered, and concentrated under reduced pressure. The crude product was purified by a chromatographic technique.

##### (4S,4aS,4bS,8S,8aS,10aS)-8a-(Bromomethyl)-4-hydroxy-8-isopropyl-4,10a-dimethyl-1-(phenylthio)-1,4,4a,4b,7,8,8a,9,10,10a-decahydrophenanthren-3(2H)-one (**1**)

The above was prepared according to general procedure GP1 from sphaerococcenol A (21.0 mg, 0.055 mmol), methanol (1.8 mL), 4-methoxybenzenethiol (13.6 µL, 2.0 equiv, 0.110 mmol), and potassium *tert*-butoxide (9.2 mg, 1.5 equiv, 0.082 mmol). The reaction was stirred for 40 min. The crude product was purified by silica gel chromatography (eluent: *n*-hexane/ethyl acetate (9:1)) to afford the product as a beige, amorphous solid (15.0 mg, 56%). ^1^H NMR (400 MHz, CDCl_3_) δ 7.39 (d, *J* = 8.7 Hz, 2H), 6.87 (d, *J* = 8.8 Hz, 1H), 5.99 (d, *J* = 10.4 Hz, 1H), 5.78–5.70 (m, 1H), 4.01 (d, *J* = 10.8 Hz, 1H), 3.81 (s, 3H), 3.78 (dd, *J* = 11.0, 1.8 Hz, 1H), 3.47 (s, 1H), 3.25 (dd, *J* = 7.3, 3.2 Hz, 1H), 2.93–2.91 (m, 2H), 2.80–2.72 (m, 1H), 2.50–2.41 (m, 2H), 2.19–2.14 (m, 1H), 2.03 (s, 1H), 2.00–1.92 (m, 1H), 1.77 (d, *J* = 6.0 Hz, 1H), 1.68 (s, 1H), 1.56 (dt, *J* = 13.9, 3.7 Hz, 1H), 1.44 (s, 3H), 1.15–1.08 (m, 1H), 0.95 (d, *J* = 6.0 Hz, 6H), 0.89 (d, *J* = 6.8 Hz, 3H). ^13^C NMR (101 MHz, CDCl_3_) δ 216.9, 159.9, 135.4, 128.7, 128.0, 125.3, 115.1, 76.5, 57.8, 55.5, 45.1, 42.0, 41.3, 40.3, 40.2, 40.0, 35.8, 31.9, 31.3, 26.0, 25.9, 25.3, 22.7, 19.9, 19.4. HRMS (ESI+): exact mass calculated for [M + H]^+^ (C_27_H_37_BrO_3_S) requires *m*/*z* 521.1720, found *m*/*z* 521.1718.

##### (4S,4aS,4bS,8S,8aS,10aS)-8a-(Bromomethyl)-4-hydroxy-8-isopropyl-4,10a-dimethyl-1-(propylthio)-1,4,4a,4b,7,8,8a,9,10,10a-decahydrophenanthren-3(2H)-one (**2** and **3**)

The above was prepared according to general procedure GP1 from sphaerococcenol A (20.7 mg, 0.054 mmol), methanol (1.4 mL), propanethiol (9.8 µL, 2.0 equiv, 0.108 mmol), and potassium tert-butoxide (9.1 mg, 1.5 equiv, 0.081 mmol). The reaction was stirred for 4 h. The crude product was purified by silica gel preparative thin-layer chromatography (eluent: 100% toluene). The mixture of the epimers was isolated as a translucid oil (11.3 mg, 46%, diastereomeric ratio 1.0:1.55) and was submitted to reverse-phase semi-preparative HPLC (water–acetonitrile 50–95%) obtaining 2.3 mg of compound **2** and 3.7 mg of compound **3**.

Compound **2**: ^1^H NMR (400 MHz, CDCl_3_) δ 5.98 (d, 1H), 5.75–5.70 (m, 1H), 3.83 (d, J = 10.7 Hz, 1H), 3.74 (dd, *J* = 10.7, 1.8 Hz, 1H), 3.28 (dd, *J* = 18.1, 10.4 Hz, 1H), 3.02 (dd, *J* = 10.4, 7.3 Hz, 1H), 2.83–2.69 (m, 2H), 2.56 (t, *J* = 7.3 Hz, 2H), 2.20–2.08 (m, 1H), 2.03 (d, *J* = 9.2 Hz, 1H), 1.98–1.91 (m, 2H), 1.86 (d, *J* = 12.2 Hz, 1H), 1.79–1.73 (m, 1H), 1.72–1.64 (m, 1H), 1.63 (dd, *J* = 7.3, 2.2 Hz, 1H), 1.58 (s, 10H), 1.53 (t, *J* = 3.4 Hz, 1H), 1.31 (s, 4H), 1.01 (t, *J* = 7.3 Hz, 3H), 0.95 (d, *J* = 6.8 Hz, 3H), 0.91–0.83 (m, 7H), 0.81 (s, 3H). ^13^C NMR (101 MHz, CDCl_3_) δ 215.7, 128.7, 128.2, 76.0, 53.3, 51.6, 42.3, 42.0, 40.3, 40.1, 40.0, 36.0, 34.8, 33.9, 31.7, 25.9, 25.8, 25.0, 23.2, 22.7, 19.3, 13.7, 13.2. HRMS (ESI+): exact mass calculated for [M + H]^+^ (C_23_H_37_BrO_2_S) requires *m*/*z* 457.1770, found *m*/*z* 457.1772.

Compound **3**: ^1^H NMR (400 MHz, CDCl_3_) δ 5.95 (d, *J* = 10.5 Hz, 1H), 5.74–5.69 (m, 1H), 3.97 (d, *J* = 10.7 Hz, 1H), 3.73 (dd, *J* = 10.7, 1.9 Hz, 1H), 3.16 (dd, *J* = 19.2, 9.0 Hz, 1H), 2.91 (dd, *J* = 19.2, 1.8 Hz, 1H), 2.79 (dd, *J* = 9.0, 1.9 Hz, 1H), 2.76–2.68 (m, 1H), 2.60 (t, *J* = 7.3 Hz, 2H), 2.41–2.33 (m, 2H), 2.18–2.10 (m, 1H), 2.03 (d, *J* = 14.6 Hz, 1H), 1.95 (qd, *J* = 6.9, 2.3 Hz, 1H), 1.76 (d, *J* = 6.0 Hz, 2H), 1.70–1.52 (m, 3H), 1.38 (s, 3H), 1.08 (d, *J* = 4.4 Hz, 1H), 1.02 (t, *J* = 7.3 Hz, 3H), 0.95 (d, *J* = 6.9 Hz, 3H), 0.93 (s, 3H), 0.89 (d, *J* = 6.8 Hz, 3H). ^13^C NMR (101 MHz, CDCl_3_) δ 217.1, 128.8, 128.0, 76.5, 53.3, 45.0, 42.8, 42.0, 40.5, 40.2, 39.8, 36.0, 35.8, 31.8, 31.4, 26.0, 25.9, 25.3, 23.2, 22.7, 19.8, 19.4, 13.7. HRMS (ESI+): exact mass calculated for [M + H]^+^ (C_23_H_37_BrO_2_S) requires *m*/*z* 457.1770, found *m*/*z* 457.1772.

##### (4S,4aS,4bS,8S,8aS,10aS)-1-(Benzylthio)-8a-(bromomethyl)-4-hydroxy-8-isopropyl-4,10a-dimethyl-1,4,4a,4b,7,8,8a,9,10,10a-decahydrophenanthren-3(2H)-one (**4**)

The above was prepared according to general procedure GP1 from sphaerococcenol A (21.0 mg, 0.055 mmol), methanol (1.4 mL), benzylthiol (12.9 µL, 2.0 equiv, 0.110 mmol), and potassium tert-butoxide (9.2 mg, 1.5 equiv, 0.082 mmol). The reaction was stirred for 22 h. The crude product was purified by two consecutive silica gel preparative thin-layer chromatography runs (eluent: *n*-hexane–ethyl acetate (9.5:0.5)) to afford the product as a translucid oil (11.0 mg, 41%). ^1^H NMR (300 MHz, CDC_l3_) δ 7.37–7.27 (m, 5H), 5.95 (d, *J* = 10.4 Hz, 1H), 5.71 (ddt, *J* = 10.4, 5.3, 2.9 Hz, 1H), 3.79 (d, *J* = 3.2 Hz, 2H), 3.70 (d, *J* = 1.5 Hz, 2H), 3.49 (s, 1H), 3.09 (dd, *J* = 17.7, 10.2 Hz, 1H), 2.87 (dd, *J* = 10.2, 6.8 Hz, 1H), 2.81–2.63 (m, 1H), 2.20–2.05 (m, 1H), 2.04–1.89 (m, 1H), 1.80–1.72 (m, 2H), 1.67 (dd, *J* = 13.9, 4.0 Hz, 1H), 1.49 (t, *J* = 3.6 Hz, 1H), 1.26 (s, 3H), 1.24 (s, 3H), 0.94 (d, *J* = 6.8 Hz, 3H), 0.88 (d, *J* = 6.8 Hz, 3H), 0.81 (s, 3H). ^13^C NMR (101 MHz, CDCl_3_) δ 215.5, 137.8, 129.0, 128.8, 128.6, 128.2, 127.6, 76.0, 52.2, 51.5, 42.0, 41.9, 40.3, 40.0, 39.9, 36.9, 35.9, 33.8, 31.6, 29.8, 25.9, 25.8, 25.0, 22.7, 19.3, 13.5. HRMS (ESI+): exact mass calculated for [M − H_2_O]^+^ (C_27_H_37_BrO_2_S) requires *m*/*z* 487.1665, found *m*/*z* 487.1668.

#### 4.1.4. Reduction of Sphaerococcenol A

In a round-bottom flask equipped with a stir bar, flame-dried and purged under an argon atmosphere, a solution of sphaerococcenol A (16.0 mg, 0.041 mmol) was prepared in distilled methanol (1.4 mL). Then, sodium borohydride (2 equiv, 0.082 mmol) was added and the mixture was left reacting for 40 min. A saturated solution of ammonium chloride (6 mL) was added to the mixture and extracted with dichloromethane (4 × 4 mL). The organic phases were combined and dried with anhydrous magnesium sulphate, filtered, and concentrated under reduced pressure. The crude product was purified by silica gel column chromatography (eluent: *n*-hexane–ethyl acetate (7.5:2.5). The two epimers were isolated as pale oils (6.0 mg of each epimer, 76% overall yield).

Compound **5**: ^1^H NMR (400 MHz, CDCl_3_) δ 6.04 (d, *J* = 10.6 Hz, 1H), 5.69–5.63 (m, 1H), 3.99 (d, *J* = 10.5 Hz, 1H), 3.65 (dd, *J* = 10.5, 2.3 Hz, 1H), 3.30 (d, *J* = 11.4 Hz, 1H), 2.94–2.89 (m, 1H), 2.20–2.09 (m, 1H), 2.07 (s, 1H), 2.02–1.88 (m, 2H), 1.88–1.81 (m, 2H), 1.78–1.72 (m, 2H), 1.61 (qt, *J* = 5.6, 2.7 Hz, 3H), 1.47 (dd, *J* = 4.4, 2.5 Hz, 1H), 1.43 (s, 4H), 1.40 (t, *J* = 3.5 Hz, 1H), 1.32 (d, *J* = 3.5 Hz, 1H), 1.29 (d, *J* = 5.5 Hz, 1H), 1.23 (q, *J* = 2.3 Hz, 1H), 1.19 (q, *J* = 2.2 Hz, 1H), 1.13 (s, 3H), 0.96 (d, *J* = 6.8 Hz, 3H), 0.91 (d, *J* = 6.9 Hz, 3H). ^13^C NMR (101 MHz, CDC_l3_) δ 129.6, 126.0, 78.3, 73.8, 49.0, 42.8, 41.2, 41.0, 40.1, 38.0, 37.1, 36.3, 31.0, 26.4, 26.0, 25.0, 22.1, 20.1, 19.3. HRMS (ESI+): exact mass calculated for [M − H_2_O]^+^ (C_20_H_33_BrO_2_) requires *m*/*z* 367.1631, found *m*/*z* 367.1632.

Compound **6:** ^1^H NMR (300 MHz, CDCl_3_) δ 6.02 (d, *J* = 10.4 Hz, 1H), 5.72–5.65 (m, 1H), 3.99 (d, *J* = 10.6 Hz, 1H), 3.66 (dd, *J* = 10.6, 2.1 Hz, 1H), 3.61 (dd, *J* = 5.6, 3.0 Hz, 1H), 2.90–2.83 (m, 1H), 2.21–1.87 (m, 5H), 1.80 (ddd, *J* = 14.5, 5.1, 2.1 Hz, 1H), 1.74–1.70 (m, 1H), 1.65–1.57 (m, 1H), 1.56–1.46 (m, 1H), 1.43 (s, 3H), 1.37–1.18 (m, 4H), 1.16 (s, 3H), 0.96 (d, *J* = 6.8 Hz, 3H), 0.91 (d, *J* = 6.9 Hz, 3H). ^13^C NMR (75 MHz, CDCl_3_) δ 129.3, 127.3, 78.1, 77.4, 75.3, 45.6, 42.8, 41.4, 41.1, 38.9, 36.2, 36.1, 35.8, 30.7, 26.2, 26.1, 25.4, 25.0, 22.2, 21.0, 19.8. HRMS (ESI+): exact mass calculated for [M − H_2_O]^+^ (C_20_H_33_BrO_2_) requires *m*/*z* 367.1631, found *m*/*z* 367.1631.

#### 4.1.5. Studies on Thiol Attack: Thiol-Michael Addition versus SN2

##### Sphaerococcenol A with Nucleophile in Excess (20.0 equiv)

The above was prepared according to general procedure GP1 from sphaerococcenol A (50.0 mg, 0.131 mmol), methanol (5 mL), 4-methoxybenzenethiol (32.5 µL, 20.0 equiv, 0.262 mol), and potassium *tert*-butoxide (15.0 equiv, 0.197 mol). The reaction was stirred for 3 h. The crude product was purified by silica gel chromatography (eluent: *n*-hexane–ethyl acetate (9:1)) to afford the product as a beige, amorphous solid (37.5 mg, 55%).

##### 1-Bromo-2,2-d’imethylpropane as Surrogate Molecule

The above was prepared according to general procedure GP1 from a solution of 1-bromo-2,2-dimethylpropane (17 µL, 0.132 mmol), methanol (4 mL), 4-methoxybenzenethiol (32.7 µL, 2.0 equiv, 0.265 mmol), and potassium *tert*-butoxide (22.3 mg, 1.5 equiv, 0.198 mmol). The reaction was stirred for 24 h. No reaction occurred.

### 4.2. Cell Culture Conditions

Three malignant cell lines (between passages 10 and 25) obtained from the DMSZ-German Collection of Microorganisms and Cell Cultures GmbH biobank were used: A549 (lung carcinoma), DU-145 (prostate carcinoma), and MCF-7 (breast adenocarcinoma). MCF-7 (ACC-115) and DU-145 (ACC-261) cells were maintained in RPMI 1640 medium (Merck, Darmstadt, Germany) supplemented with 10% FBS (fetal bovine serum, Biowest, Riverside, MO, USA) and 1% antimycotic and antibiotic solution (Biowest, Nuaillé, France). A549 (ACC-107) cells were maintained in DMEM:F12 medium (Merck, Darmstadt, Germany) and also supplemented with 10% FBS (Biowest, Riverside, MO, USA) and a 1% solution of antimycotic and antibiotic (Biowest, Nuaillé, France). FL83B hepatocytes (FL83B; ATCC: CLR-2390) were used as the non-malignant cell line. The cells were cultured in F-12K medium supplemented with FBS 10% (Biowest, Riverside, MO, USA) and 1% solution of antimycotic and antibiotic (Biowest, Nuaillé, France).

Upon reaching a confluency of about 80%, subculturing was initiated. The cells were lifted from the plate with a 1% trypsin solution (Sigma-Aldrich, St. Louis, MO, USA), and the neutralization was accomplished by adding culture medium. The cells were then subjected to centrifugation at 290× *g* for 5 min at room temperature. The supernatant was removed and cells were resuspended in fresh medium with a 1:8 split. Subsequently, they were planted in 25 cm^2^ T-flasks and placed in the incubator at a temperature of 37 °C and 5% CO_2_.

#### 4.2.1. Cytotoxicity of Compounds

The effects of sphaerococcenol A and its analogues (**1**–**6**) on the viability of malignant cell lines were studied through three distinct assays, 3-(4,5-dimethylthiazol-2-yl)-2,5-diphenyltetrazolium bromide (MTT), lactate dehydrogenase (LDH) activity, and calcein-AM, targeting different cellular biomarkers. Untreated cells with DMSO (<0.2%) were used as control, and saponin (0.4 mg/mL) (Sigma, Darmstadt, Germany) was used as the positive control for cell death.

#### 4.2.2. 3-(4,5-Dimethylthiazol-2-yl)-2,5-diphenyltetrazolium Bromide (MTT)

The cells were incubated with compounds at different concentrations (1–100 µM) for 24 h. After the 24 h was over, cells were washed with PBS buffer (pH = 7.4) and incubated for 60 min with the MTT solution (1.2 mM) previously prepared in a fresh medium. The MTT solution was then removed, and 100 µL of dimethyl sulfoxide (DMSO) was added to dissolve the formazan crystals. The results were expressed as the half-maximal inhibitory concentration (IC_50_) after reading the absorbance at 570 nm using a microplate reader (Synergy H1, BioTek Instruments, USA). The selectivity index (SI) was calculated as the ratio of the IC_50_ value for a normal cell line to the IC_50_ value for the corresponding tumor cell line (SI = IC_50_ of non-malignant cell line/IC_50_ of malignant cell line), where an SI ≥ 2 of a compound represents a selective toxicity towards malignant cells, while an SI value < 2 is considered generally toxic [35].

#### 4.2.3. Lactate Dehydrogenase (LDH) Activity

The cytotoxicity was measured by the LDH cytotoxicity assay kit (Pierce™ LDH Cytotoxicity Assay Kit; ThermoScientifc, Rockford, IL, USA) according to the manufacturer’s instructions. Cell death was quantified and expressed as the percentage of control untreated cells.

#### 4.2.4. Calcein-AM Assay

The cell viability was evaluated through the activity of esterases that interact with the calcein-AM probe by transforming it into calcein, a molecule that emits fluorescence and is trapped in the cytoplasm of living cells. After exposure to compounds (1–100 µM; 24 h), the cells were washed with PBS buffer and loaded with 2 µM of a calcein-AM (Invitrogen, Carlsbad, CA, USA) solution, which was previously dissolved in a PBS buffer and incubated for 30 min at room temperature protected from the light. The fluorescence emitted was measured using a microplate reader (Synergy H1, BioTek Instruments, Winooski, VT, USA) at wavelengths of 485 nm (excitation) and 530 nm (emission). The cell viability was expressed in percentage of control untreated cells.

### 4.3. Levels of Reactive Oxygen Species

This assay is based on the degree of fluorescence that is generated by the reaction of carboxy-H2DCFDA, which is a non-fluorescent probe that when in contact with reactive oxygen species (ROS) becomes oxidized, emitting fluorescence. Cells were seeded in 96-well plates and further incubated with compounds (10, 30, and 100 µM) for 3 and 6 h. After that, cells were washed with PBS and incubated with 100 µL of 20 µM carboxy-H2DCFDA solution (Invitrogen, C400) for 60 min. The fluorescence was read at wavelengths of 527 nm (emission) and 495 nm (excitation) using a microplate reader (Synergy H1, BioTek Instruments, Winooski, VT, USA). The results were expressed in the percentage of control untreated cells.

### 4.4. Mitochondrial Membrane Potential

To evaluate changes in the mitochondrial membrane potential, assays were carried out with a JC-1 fluorescent probe (Molecular Probes, Eugene, OR, USA), which evaluates the membrane potential through a ratio between the number of monomers and existing aggregates. Cells were incubated with compounds (10, 30, and 100 µM) for 3 and 6 h. The measurement of JC-1 aggregates (λ excitation: 490 nm; λ emission: 590 nm) and monomers (λ excitation: 490 nm; λ emission: 530 nm) was performed using a microplate reader (Synergy H1, BioTek Instruments, Winooski, VT, USA). The results were expressed in percentage of control untreated cells.

### 4.5. Biomarkers Related to Apoptosis

#### 4.5.1. Caspase-3 Activity

The activity of Caspase-3 was determined using a commercial kit (Sigma, Casp3f, St. Louis, MO, USA) according to the manufacturer’s instructions. To carry out this assay, cells were seeded in 6-well plates and subsequently incubated with compounds (10, 30, and 100 µM) for 6 and 12 h. Cells were then washed with PBS, transferred to a microtube, and centrifuged at 3200× *g* for 5 min at 4 °C. The resulting pellet was resuspended in 50 µL of lyse buffer and then kept on ice for 20 min. After that, cells were centrifugated at 15,300× *g* for 20 min at 4 °C. The supernatant was used to quantify Caspase-3 activity and the pellet for protein quantification. The fluorescence read was performed in a microplate reader (Synergy H1, BioTek Instruments, Winooski, VT, USA) during 60 min at wavelengths of 360 nm (excitation) and 460 nm (emission). The results were expressed in percentage of control untreated cells (∆fluorescence (u.a)/mg of protein/min).

#### 4.5.2. DAPI Staining

The purpose of employing the 4′,6-Diamidino-2-Phenylindole, Dihydrochloride (DAPI) method is to investigate alterations occurring at the DNA level. DAPI serves as a fluorescent stain, exhibiting an affinity for binding to regions abundant in A and T content, which enables the visualization of genetic material condensation or fragmentation occurrences. For this assay, 6-well plates were seeded and subsequently exposed to compounds (10, 30, and 100 µM) for 12 and 24 h. After this, cells were stained with DAPI, as previously described by Silva et al. [36]. A representative image of each treatment condition was taken with a camera (AxioCam MRC-ZEISS, Jena, Germany) coupled to a fluorescence inverted microscope (ZEISS Axio, VERT. A1, Jena, Germany).

### 4.6. Data and Statistical Analysis

The data were obtained from at least three independent experiments (*n* = 3) carried out in triplicate and presented as mean ± standard error of the mean (SEM). The data were checked for normality and homoscedasticity. Data that did not meet normal distribution were analyzed by the Kruskal–Wallis non-parametric test. A one-way analysis of variance (ANOVA) with Dunnett′s multiple comparison of group means was employed to determine significant differences to the control treatment and/or a two-way ANOVA with Bonferroni’s test. All other post hoc analyses were conducted using Tukey’s test. The differences were considered significant at a level of 0.05 (*p* < 0.05). The inhibitory concentrations (IC_50_) were determined by means of the equation *y* = 100/(1 + 10(*x* − logIC_50_)). All data were analyzed using the software SPSS v28.0.0.0 (SPSS Inc., Chicago, IL, USA) and GraphPad v8.0.2 (GraphPad Software, Inc., La Jolla, CA, USA).

## 5. Conclusions

In conclusion, the data here reported correspond to the first study regarding the semi-synthesis of sphaerococcenol A analogues prepared through thiol-Michael additions and enone reduction, resulting in six new derivatives. The chemical alterations did not enhance the cytotoxic activity of the original compound, and the extra methylene group in compound **4** seems to contribute to the loss of activity. The semi-synthetic compound **1**, synthesized using 4-methoxybenzenethiol, exhibited similar cytotoxic activity of sphaerococcenol A in A549 malignant cells, which was accompanied by an increase in ROS generation and by the alterations of different biomarkers related to apoptosis cell death. This study opens new research opportunities to fully explore the potential use of this bromoditerpene as a scaffold for the synthesis of new molecules.

## Figures and Tables

**Table 1:** Determination of the IC_50_ value of sphaerococcenol A and its derivatives (**1**–**6**) in malignant (A549, DU-145, and MCF-7) and non-malignant (FL83B) cell lines after treatment for 24 h at different concentrations (1–100 µM). The effects were estimated by the MTT assay. The values in parentheses represent the confidence intervals for 95%. The selective index (SI) was also determined.

	IC_50_ (µM)						
	A549	SI	DU-145	SI	MCF-7	SI	FL83B
Sphaerococcenol A	14.99 * (12.30–18.19)	2.5	3.75 ^c,e,^* (3.17–4.44)	10.0	6.53 ^c,^* (4.43–9.57)	5.7	37.40 (31.49–44.92)
Compound **1**	18.70 (12.82–26.76)	1.8	15.82 ^c,e^ (13.33–18.92)	2.2	16.44 ^c^ (11.46–22.40)	2.1	34.56 (17.89–64.68)
Compound **2**	33.78 (27.63–41.19)	1.4	25.80 ^e^ (17.97–36.39)	1.9	70.26 * (49.57–101.7)	0.7	48.02 ^a^ (36.77–64.07)
Compound **3**	22.53 (18.36–27.56)	2.5	20.13 ^e^ (16.43–24.52)	2.8	14.31 ^c,^* (10.01–21.61)	4.0	56.69 (35.40–98.96)
Compound **4**	35.45 ^a^ (29.72–42.43)	1.1	70.11 ^a^ (39.63–145.8)	0.6	30.40 ^c^ (23.46–39.22)	1.3	40.32 (32.10–50.92)
Compound **5**	22.68 * (18.17–27.88)	3.7	18.53 ^e,^* (16.25–21.68)	4.4	31.95 ^c,^* (23.36–45.45)	2.6	82.12 ^a,b,c,d,e^ (67.32–n.d.)
Compound **6**	33.54 * (29.48–38.55)	1.9	20.46 ^e,^* (16.73–24.92)	3.1	22.19 ^c,^* (14.49–31.98)	2.9	63.29 ^b,^* (53.21–75.49)

Significant differences (*p* < 0.05) inside of the cell line group when compared with ^a^ sphaerococcenol A; ^b^ compound **1**; ^c^ compound **2**; ^d^ compound **3**; ^e^ compound **4**. Significant differences (*p* < 0.05) between different cells lines treated with the same compound when compared with * FL83B; SI = IC_50_ of FL83B/IC_50_ on the malignant cell lines. n.d., not defined.

## Data Availability

The data presented in this study are available on request from the corresponding author.

## Supplementary Materials

The following supporting information can be downloaded at: https://www.mdpi.com/article/10.3390/md22090408/s1, Table S1: Relevant ^1^H NMR spectral data of compounds **1**–**6**, and similar structures isolated from *Sphaerococcus coronopifolius*.
