# Supplementary material for: Sphaerococcenol A Derivatives: Design, Synthesis, and Cytotoxicity
Source: Mar Drugs. 2024 Sep 5;22(9):408. doi: 10.3390/md22090408 (PMC11432771; doi:10.3390/md22090408)

Supporting information

Sphaerococcenol A Derivatives: Design, Synthesis, and Cytotoxicity

Dídia Sousa[1,†], Milene A. G. Fortunato[2,†], Joana Silva[1], Mónica Pingo[1], Alice Martins[1], Carlos A. M. Afonso[2], Rui Pedrosa[1], Filipa Siopa[2,*], Celso Alves[1,*]

[1] MARE—Marine and Environmental Sciences Centre/ARNET - Aquatic Research Network, ESTM, Politécnico de Leiria, 2520-614 Peniche, Portugal;

[2] Research Institute for Medicines (iMed.ULisboa), Faculty of Pharmacy, Universidade de Lisboa, Av. Prof. Gama Pinto, 1649-003, Lisboa, Portugal;

[†]These authors contributed equally to this work

[*] Correspondence: filipasiopa@ff.ulisboa.pt (F.S.), Tel.: +351-217-946-400; celso.alves@ipleiria.pt (C.A.); Tel.: +351-262-783-607

Table of contents

Table S1. 1

Characterization - NMR spectra and HRMS 4

Table S1. Relevant ^{1}H NMR spectral data of compounds **1-6**, and similar structures isolated from *Spaherocccus coronopifolius*.

Compound	^{1}H NMR (400 MHz, $CDCl_3$)	Reported compound structure [a]	^{1}H NMR (400 MHz, $CDCl_3$)
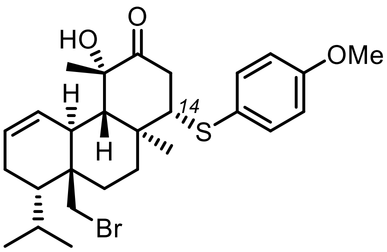 Compound **1**	H-14 (δ_H 3.25, dd, *J* = 7.3, 3.2 Hz)	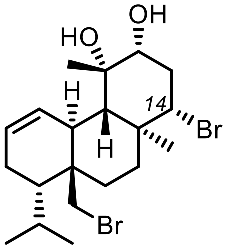	H-14 (δ_H 3.88, dd, *J* = 12.8, 3.3 Hz)
		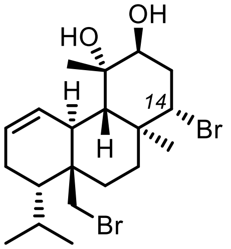	H-14 (δ_H 4.46, dd, *J* = 12.8, 3.7 Hz)
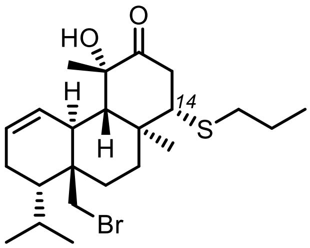 Compound **2**	H-14 (δ_H 3.02, dd, *J* = 10.4, 7.3 Hz)	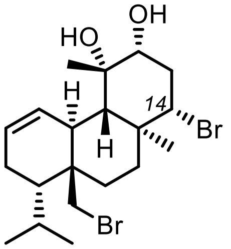	H-14 (δ_H 3.88, dd, *J* = 12.8, 3.3 Hz)
		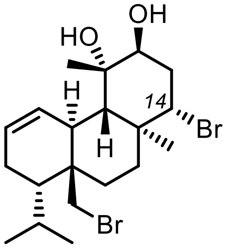	H-14 (δ_H 4.46, dd, *J* = 12.8, 3.7 Hz)
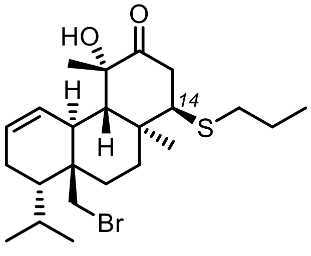 Compound **3**	H-14 (δ_H 2.79, dd, *J* = 9.0, 1.9 Hz)	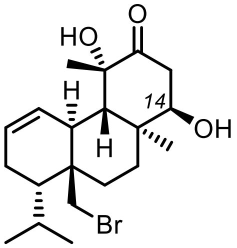	H-14 (δ_H 3.74, dd, *J* = 7.9, 1.2 Hz)

<table>
<tr>
<td rowspan="3">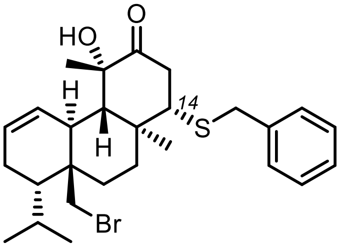
Compound **4**</td>
<td rowspan="3">H-13 (δ_H 3.09, dd, *J* = 17.7, 10.2 Hz)

H-14 (δ_H 2.87, dd, *J* = 10.2, 6.8 Hz)</td>
<td>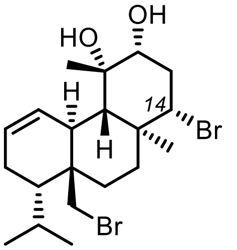</td>
<td>H-13α (δ_H 2.33, ddd, *J* = 12.8, 12.4, 11.6 Hz)

H-14 (δ_H 3.88, dd, *J* = 12.8, 3.3 Hz)</td>
</tr>
<tr>
<td>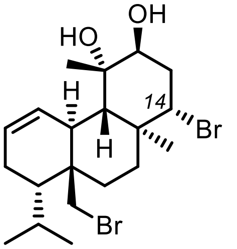</td>
<td>H-13α (δ_H 2.70, ddd, *J* = 13.7, 12.8, 12.4, 2.9 Hz)

H-14 (δ_H 4.46, dd, *J* = 12.8, 3.7 Hz)</td>
</tr>
<tr>
<td>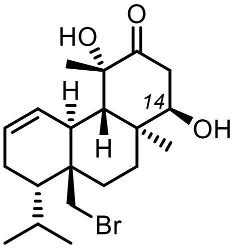</td>
<td>H-13α (δ_H 2.63, dd, *J* = 18.2, 1.2 Hz)

H-13β (δ_H 2.99, dd, *J* = 18.2, 7.9 Hz)

H-14 (δ_H 3.74, dd, *J* = 7.9, 1.2 Hz)</td>
</tr>
<tr>
<td>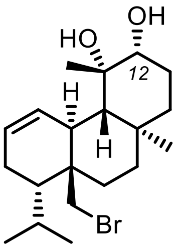
Compound **5**</td>
<td>H-12 (δ_H 3.30 d, *J* = 11.4 Hz)</td>
<td>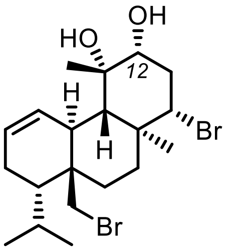</td>
<td>H-12 (δ_H 3.34, dt, *J* = 11.6, 5.4 Hz)</td>
</tr>
<tr>
<td rowspan="2">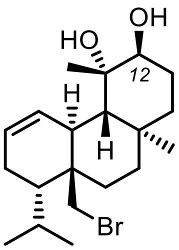
Compound **6**</td>
<td rowspan="2">H-12 (δ_H 3.61 dd, *J* = 5.6, 3.0 Hz)[[b]</td>
<td>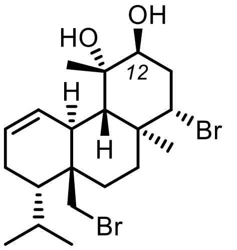</td>
<td>H-12 (δ_H 3.45, br s)</td>
</tr>
<tr>
<td>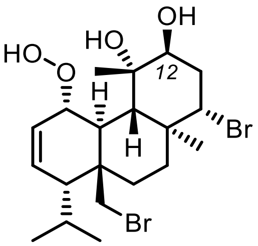</td>
<td>H-12 (δ_H 3.50, dd *J* = 3.4, 2.4 Hz)</td>
</tr>
</table>

[a] Smyrniotopoulos, V.; Quesada, A.; Vagias, C.; Moreau, D.; Roussakis, C.; Roussis, V., Cytotoxic bromoditerpenes from the red alga Sphaerococcus coronopifolius. *Tetrahedron* **2008,** 64, (22), 5184-5190.

[b] ^{1}H NMR (300 MHz, $CDCl_3$)

Characterization

^{1}H NMR (400 MHz, $CDCl_3$) of Sphaerococcenol A (SA)

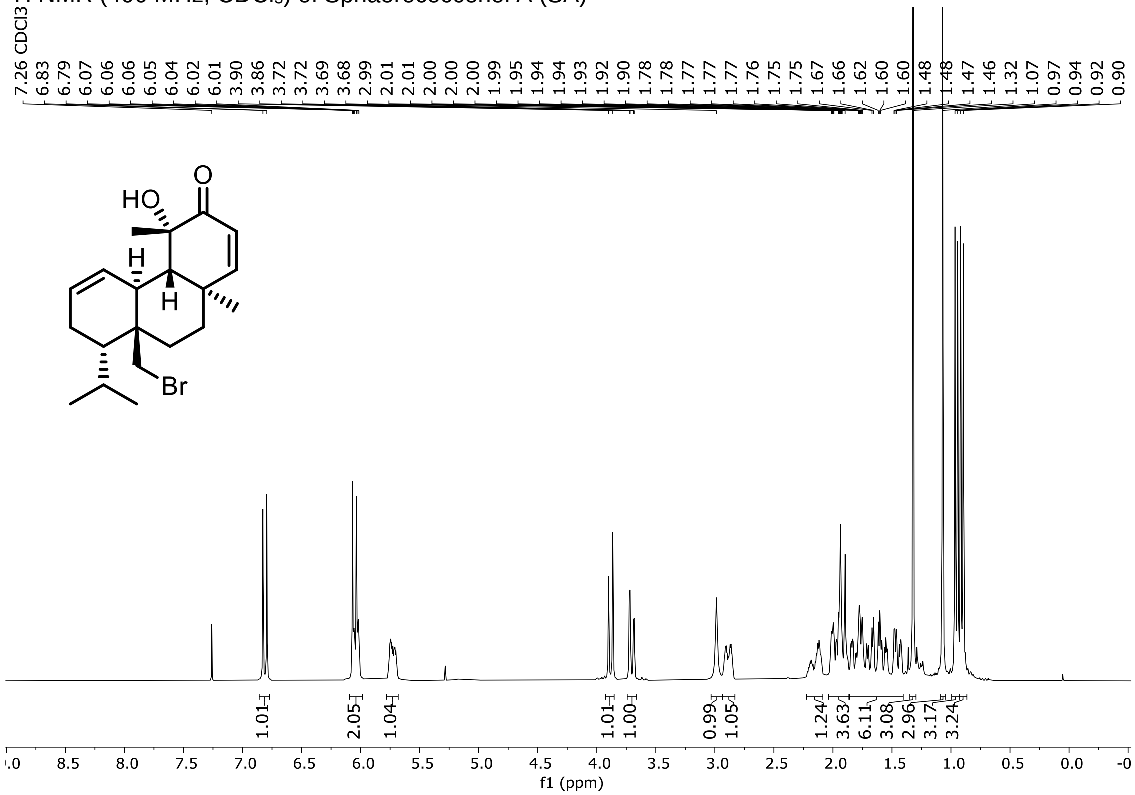

^{13}C NMR (75 MHz, $CDCl_3$) of of Sphaerococcenol A (SA)

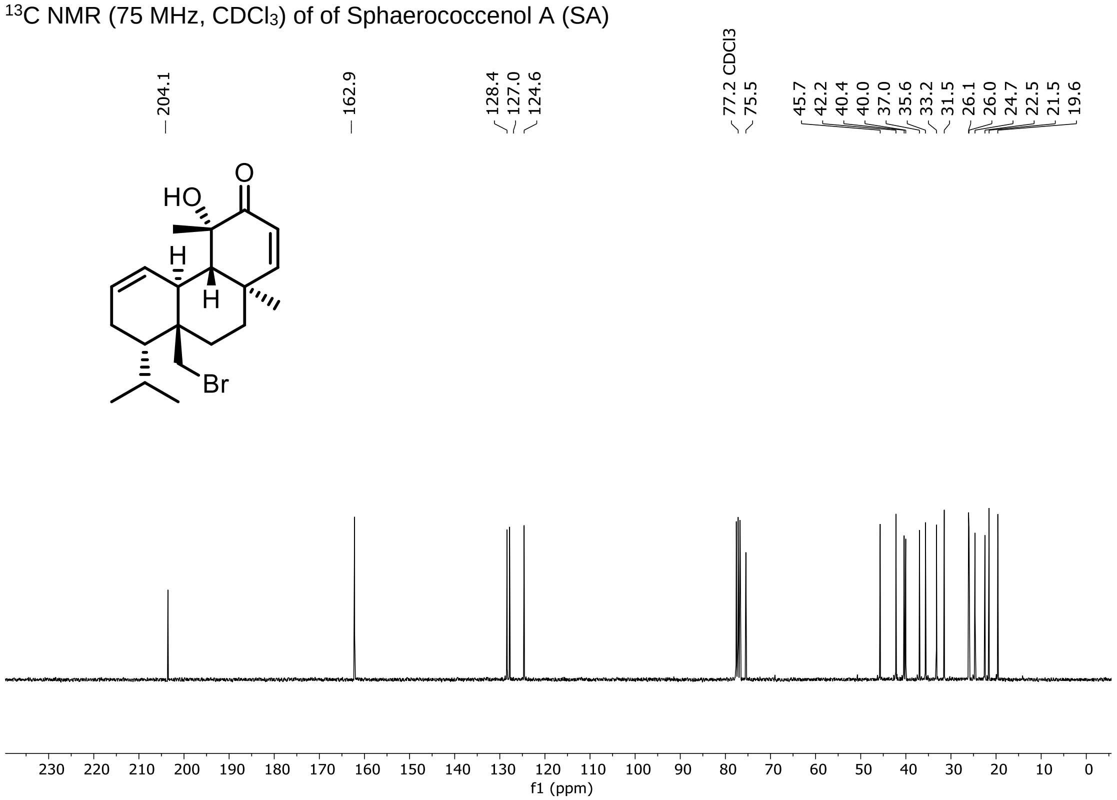

^{1}H NMR (400 MHz, $CDCl_3$) of **1**

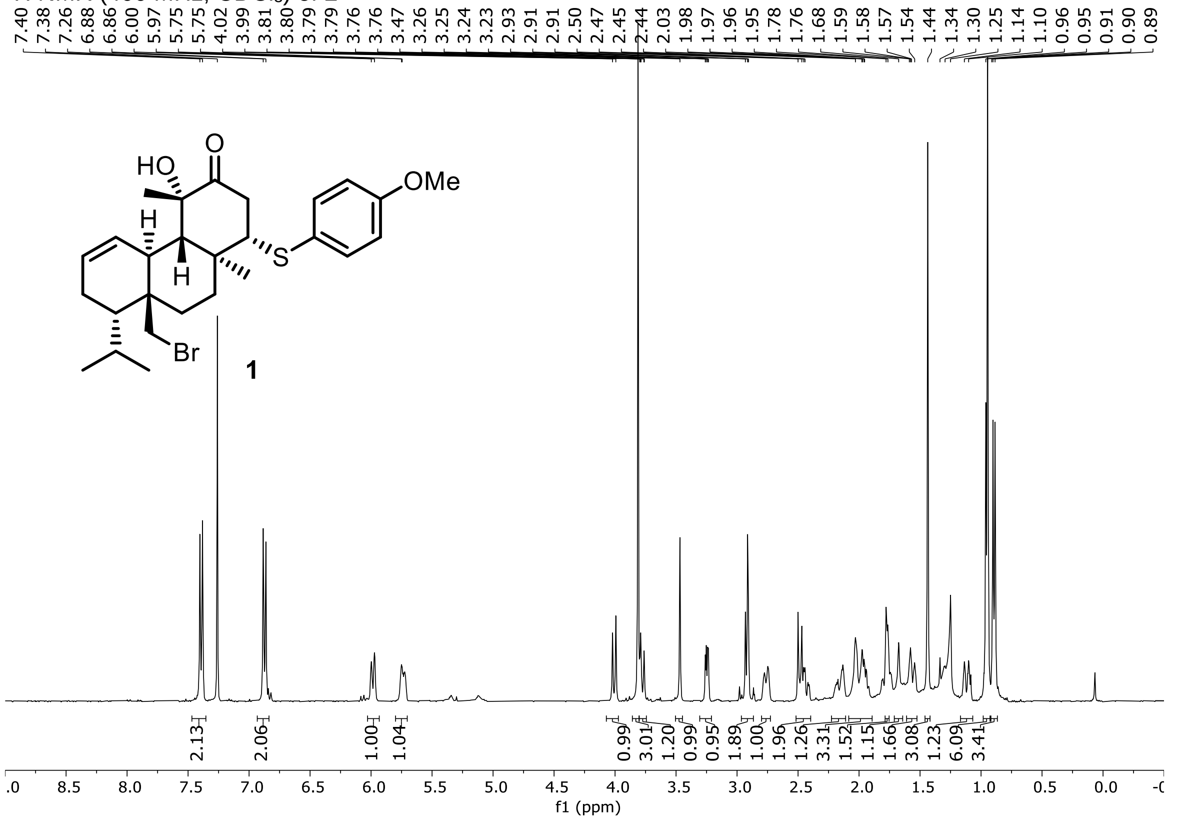

^{13}C NMR (101 MHz, $CDCl_3$) of **1**

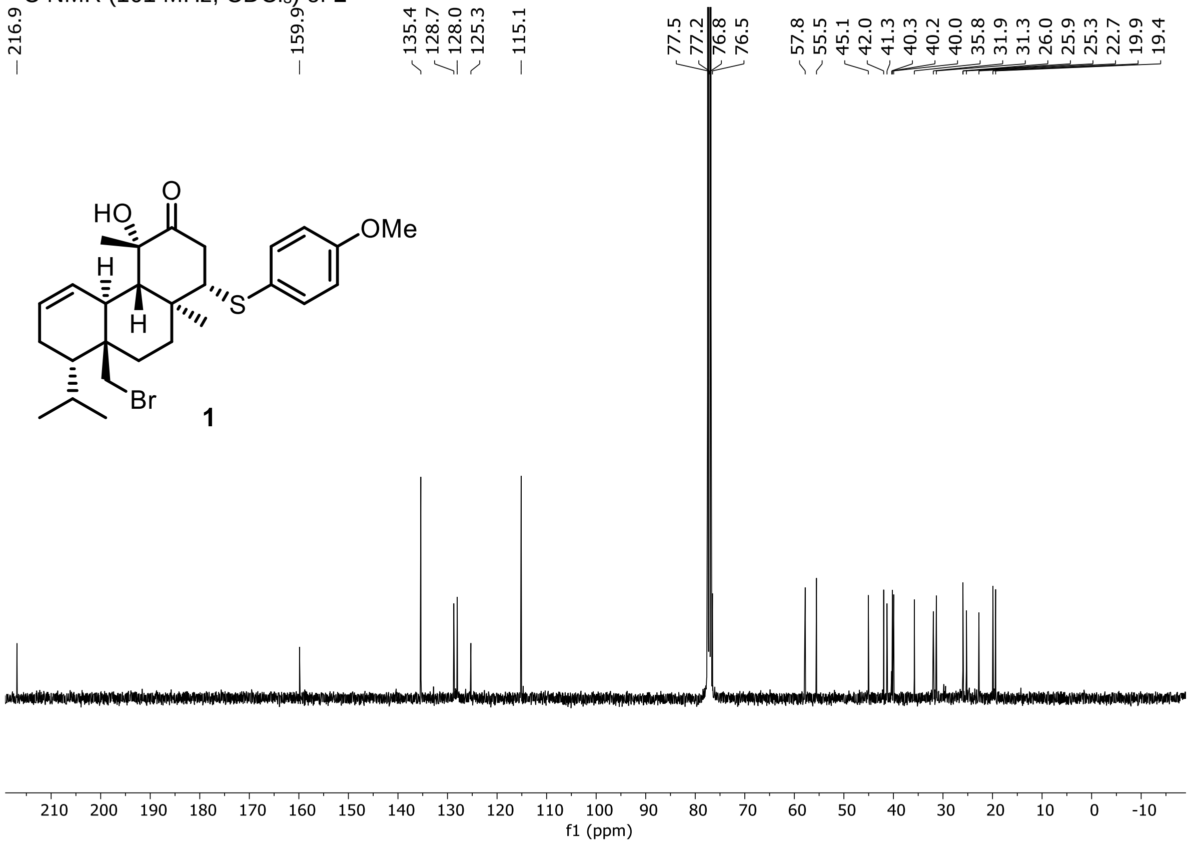

HSQC NMR ($CDCl_3$) of **1**

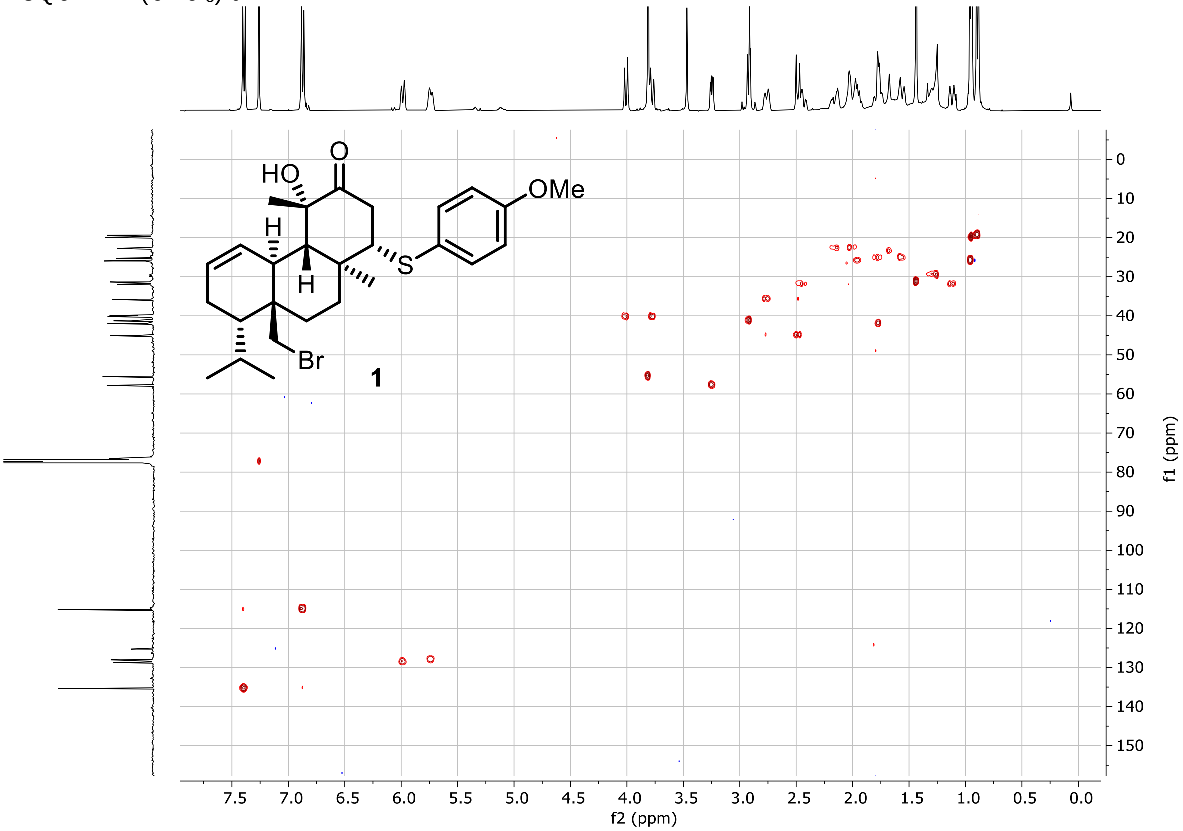

COSY NMR ($CDCl_3$) of **1**

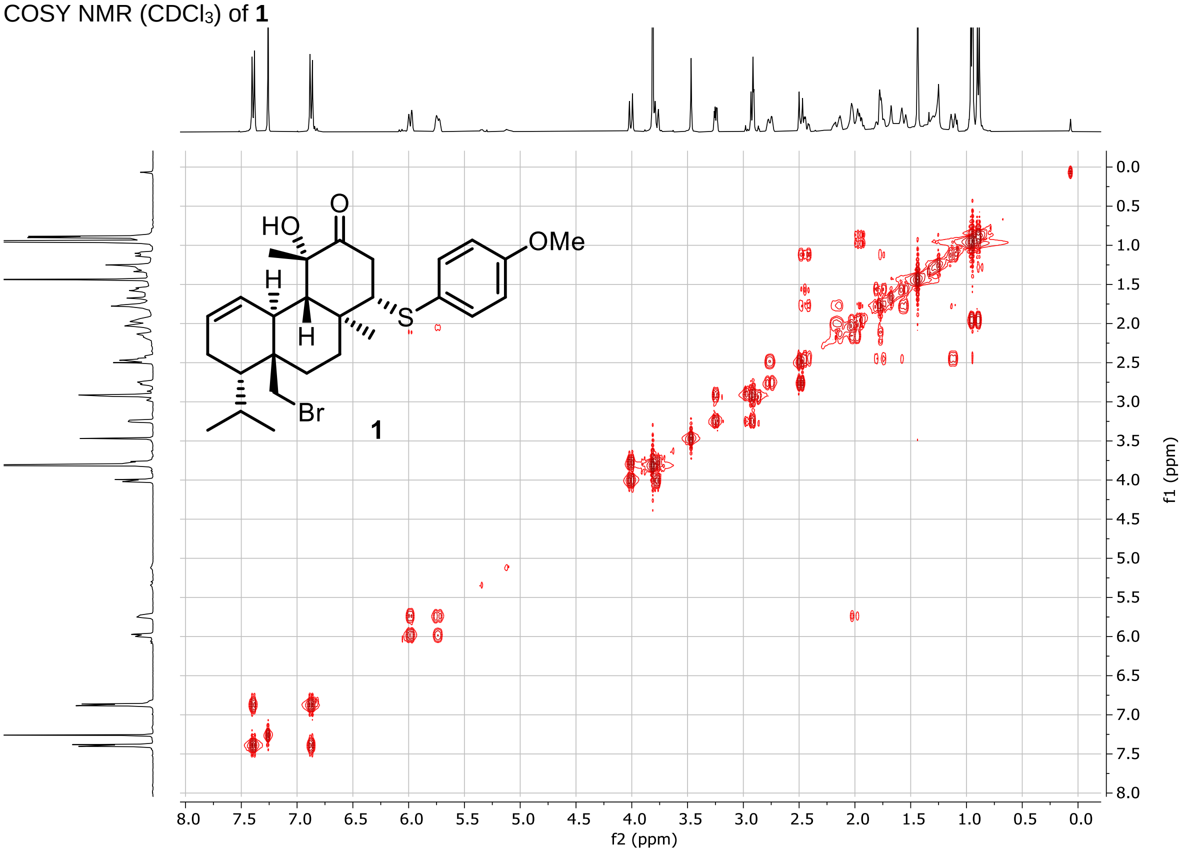

HMBC NMR ($CDCl_3$) of **1**

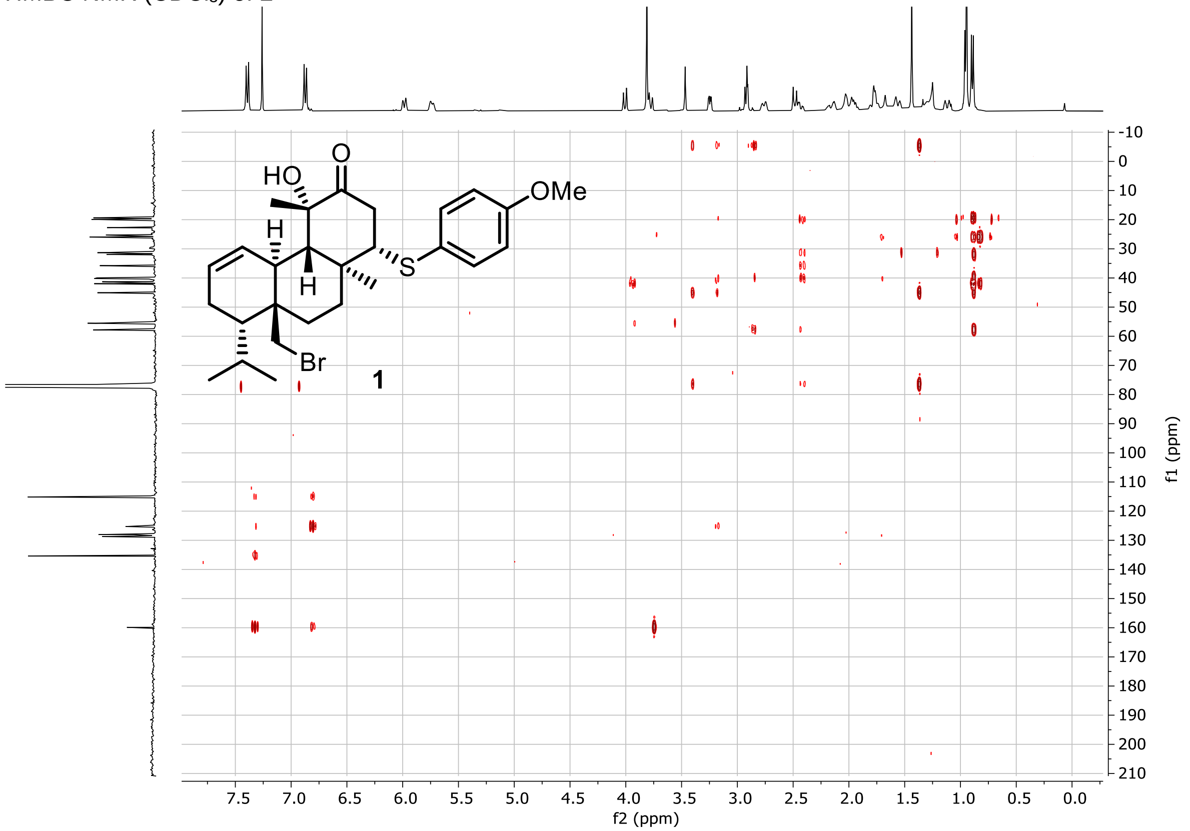

HRMS spectra of **1**

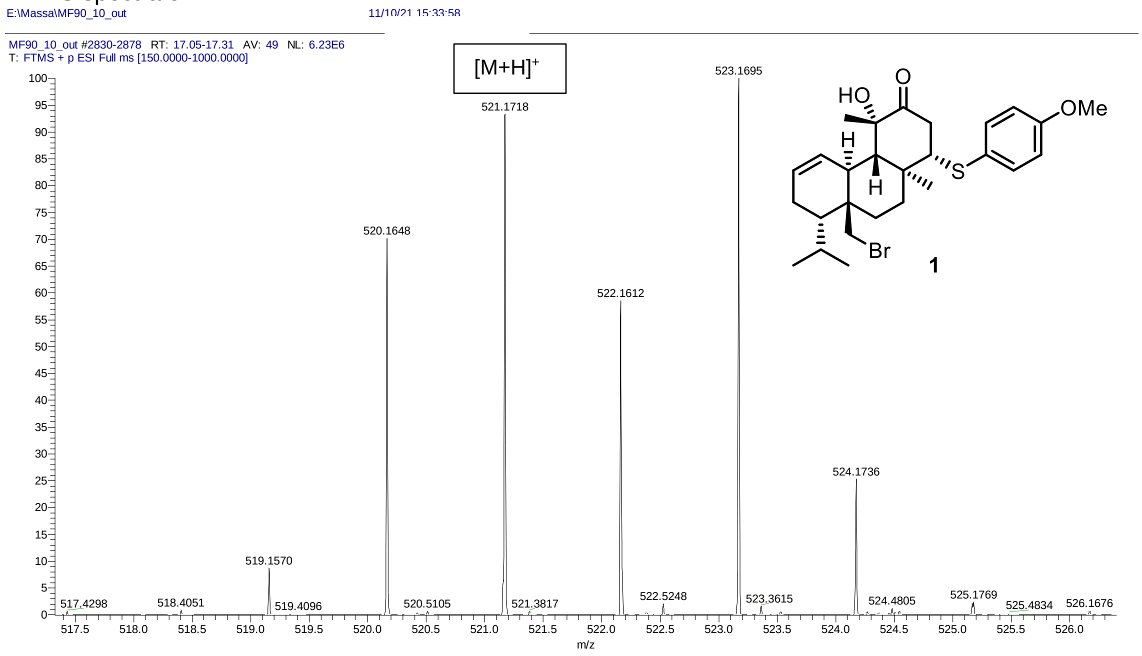

^{1}H NMR (400 MHz, $CDCl_3$) of **2**

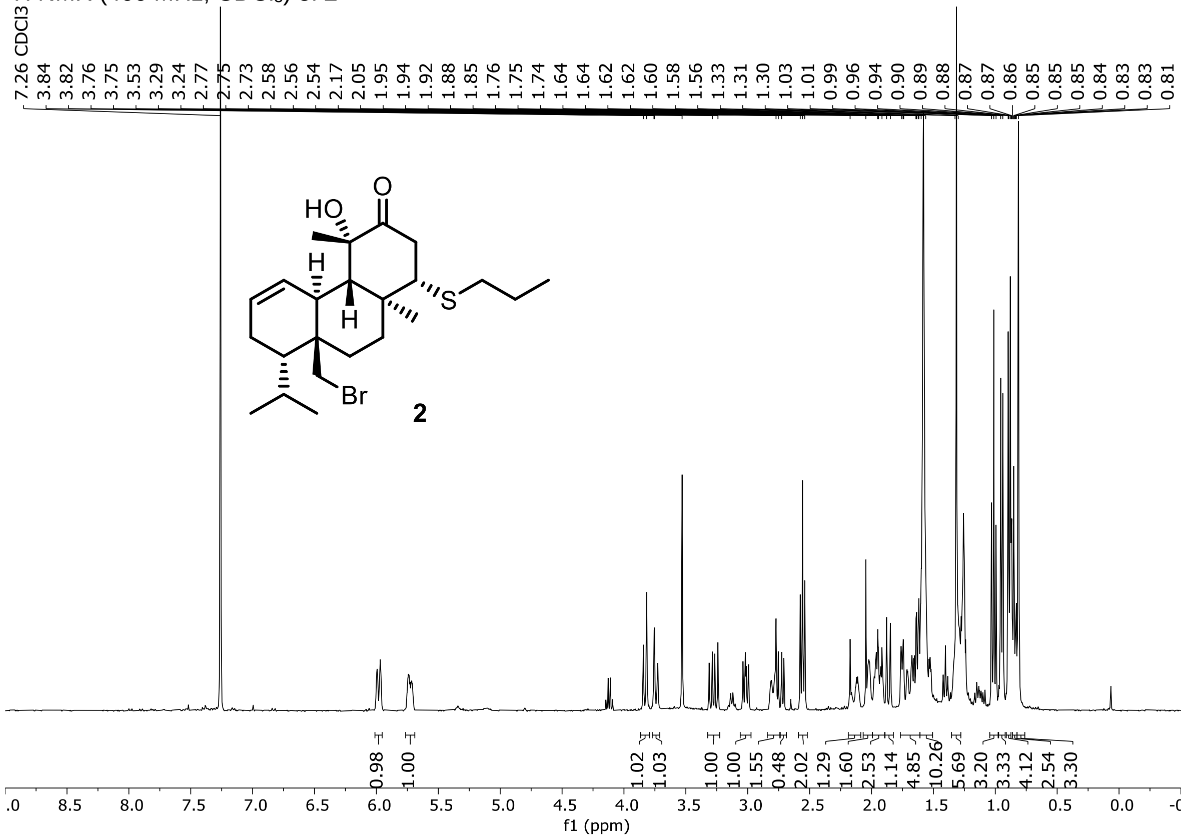

^{13}C NMR (101 MHz, $CDCl_3$) of **2**

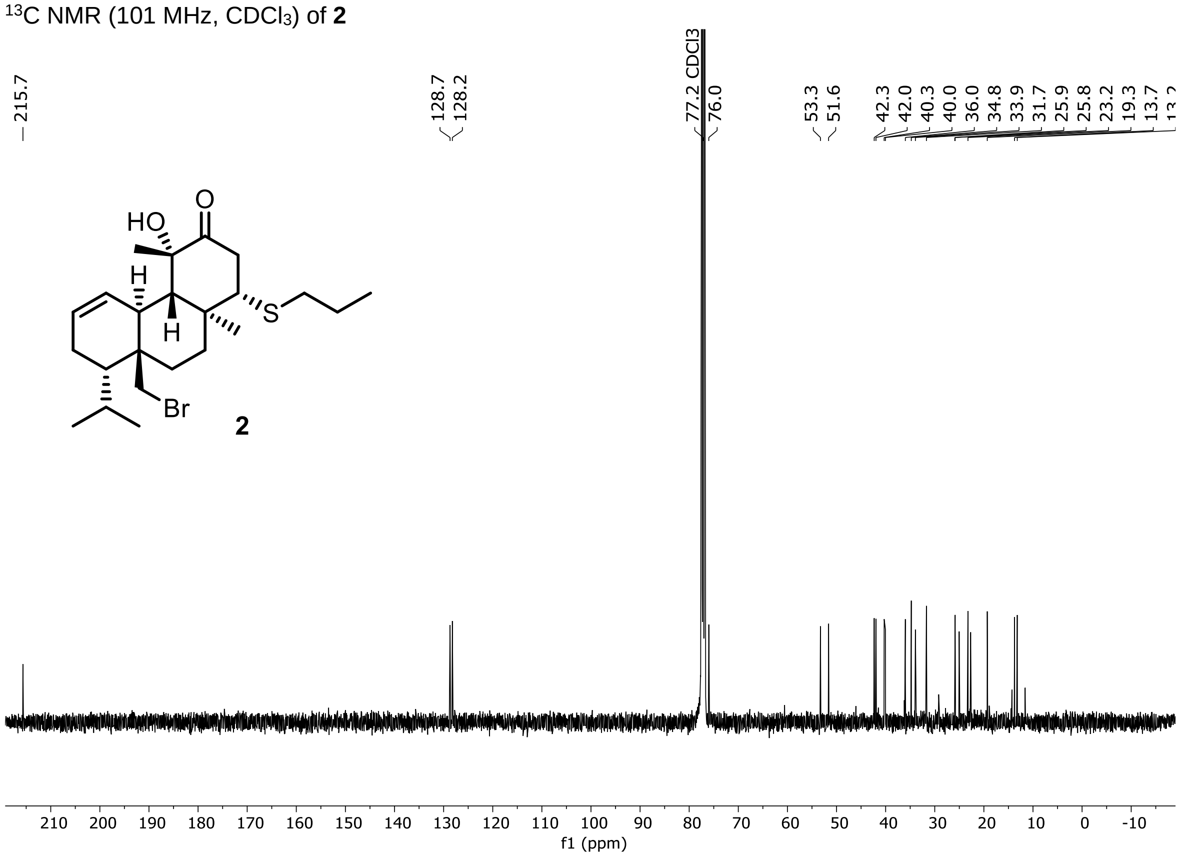

HSQC NMR ($CDCl_3$) of **2**

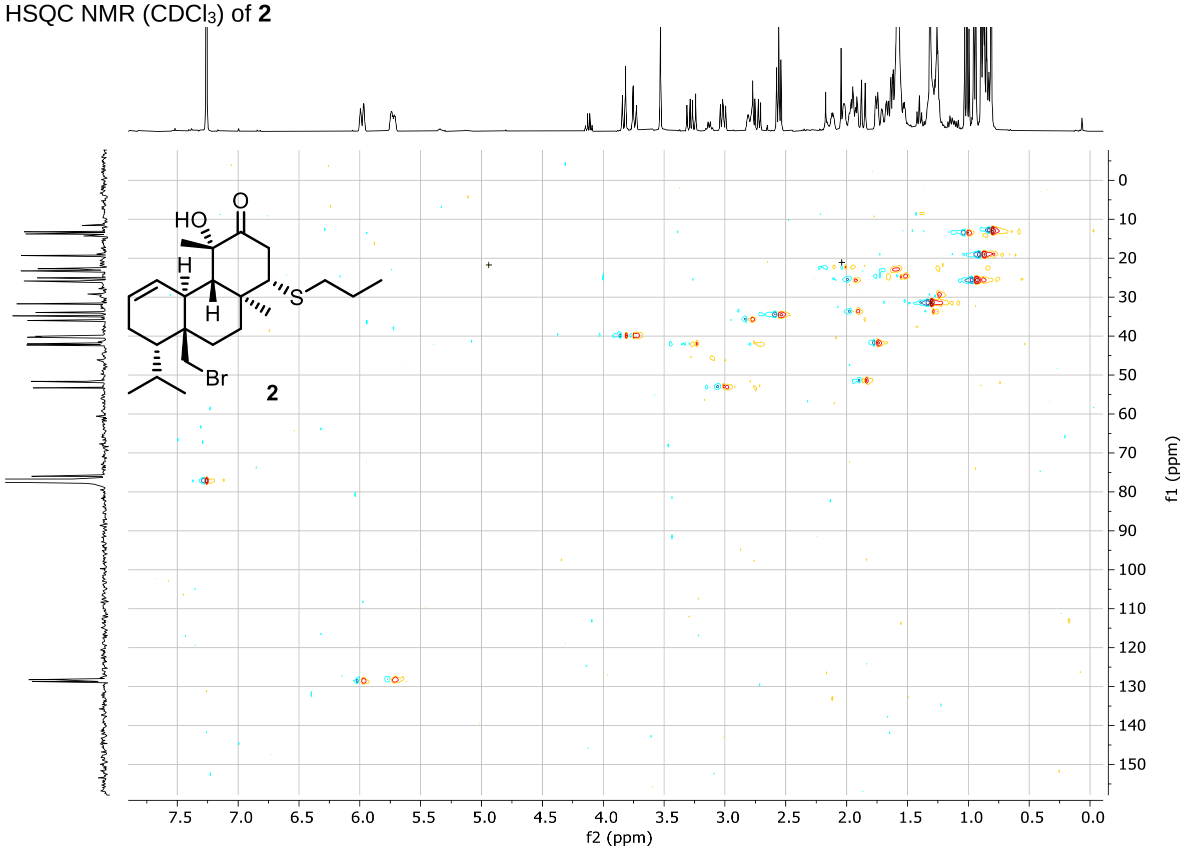

COSY NMR ($CDCl_3$) of **2**

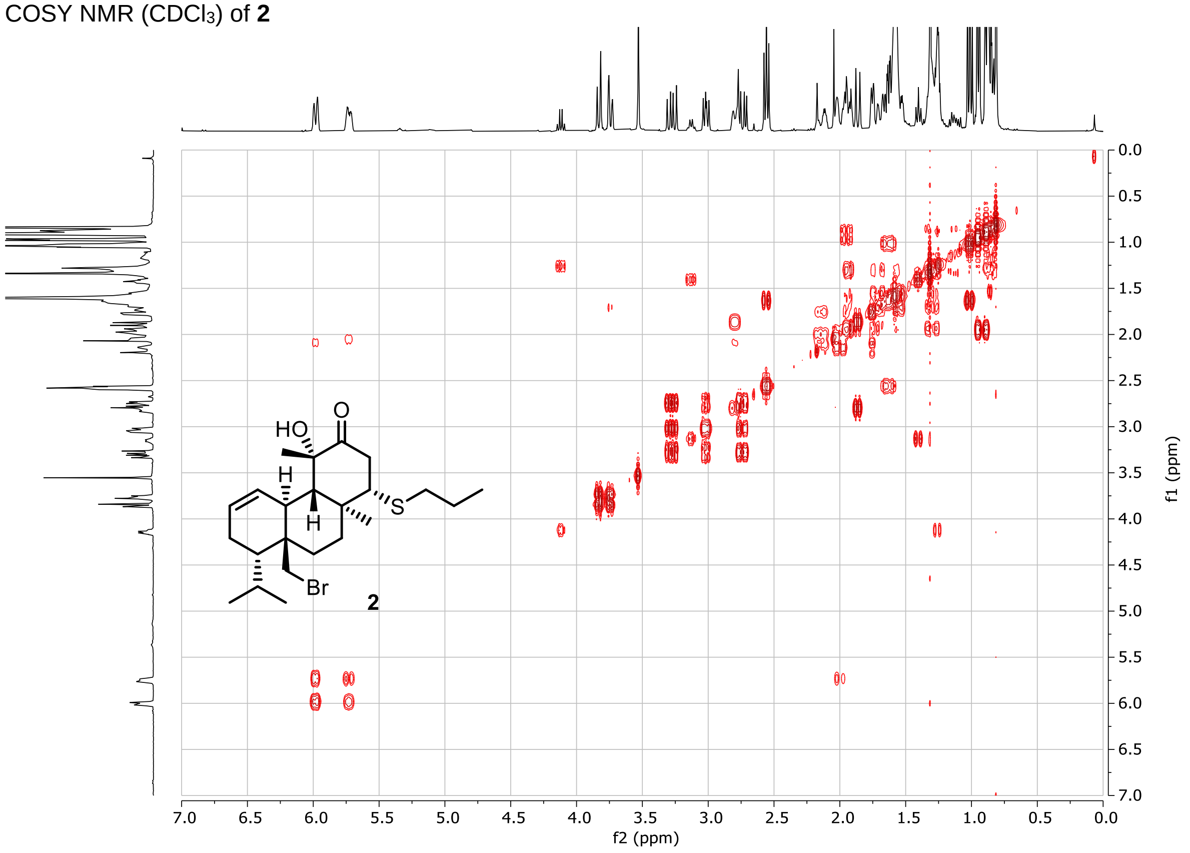

HRMS spectra of **2**

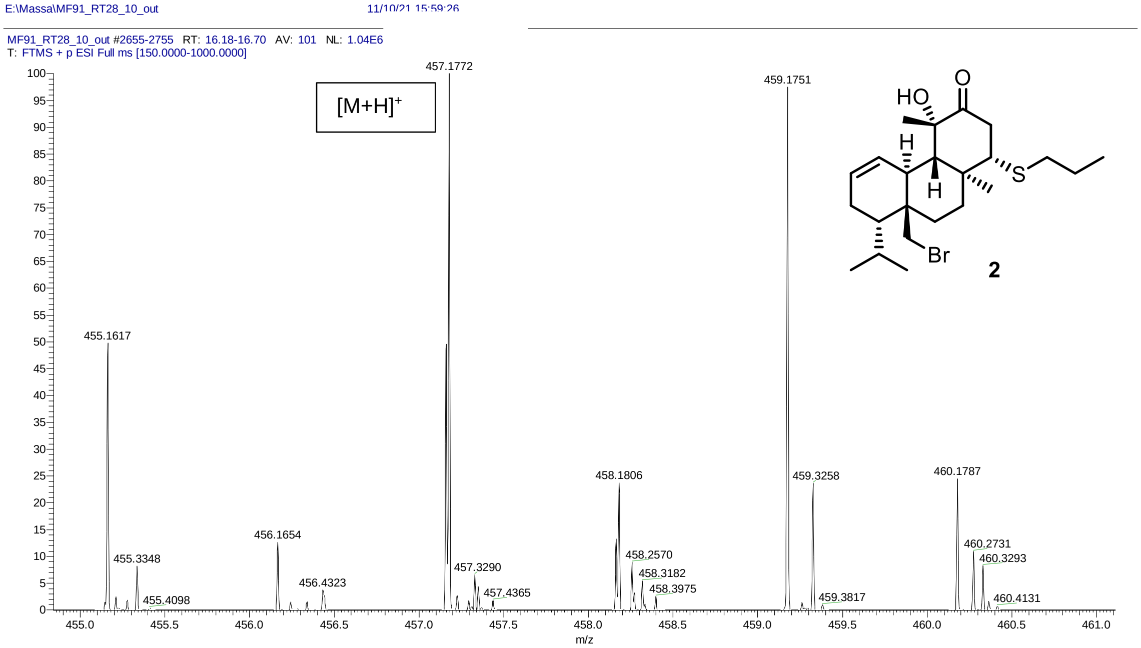

^{1}H NMR (400 MHz, $CDCl_3$) of **3**

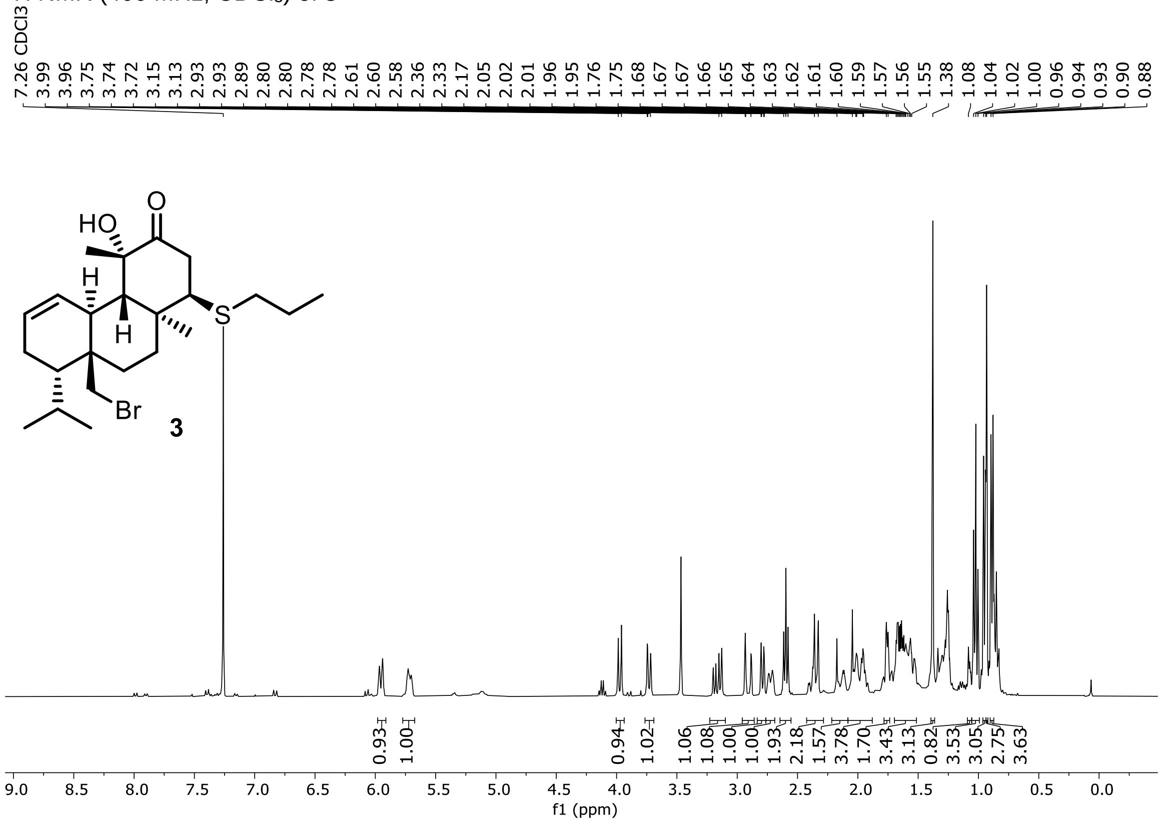

^{13}C NMR (101 MHz, $CDCl_3$) of **3**

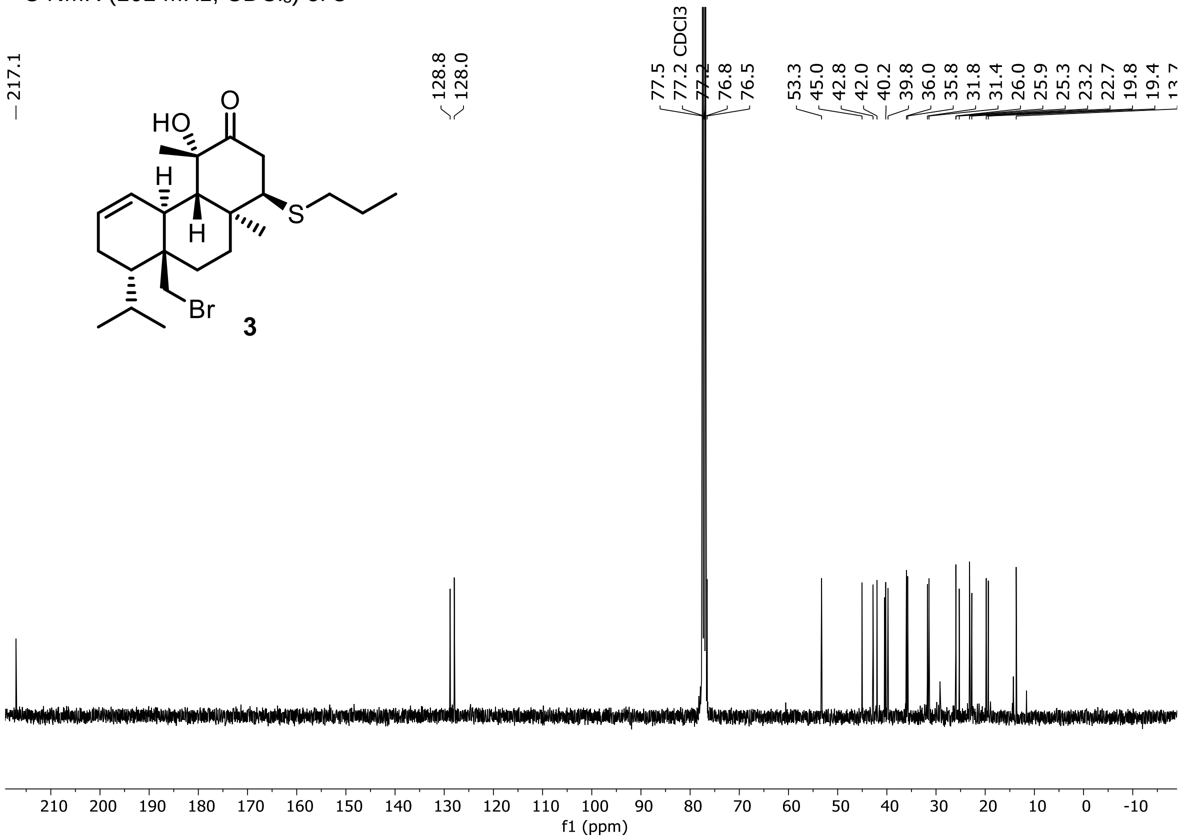

HSQC NMR ($CDCl_3$) of **3**

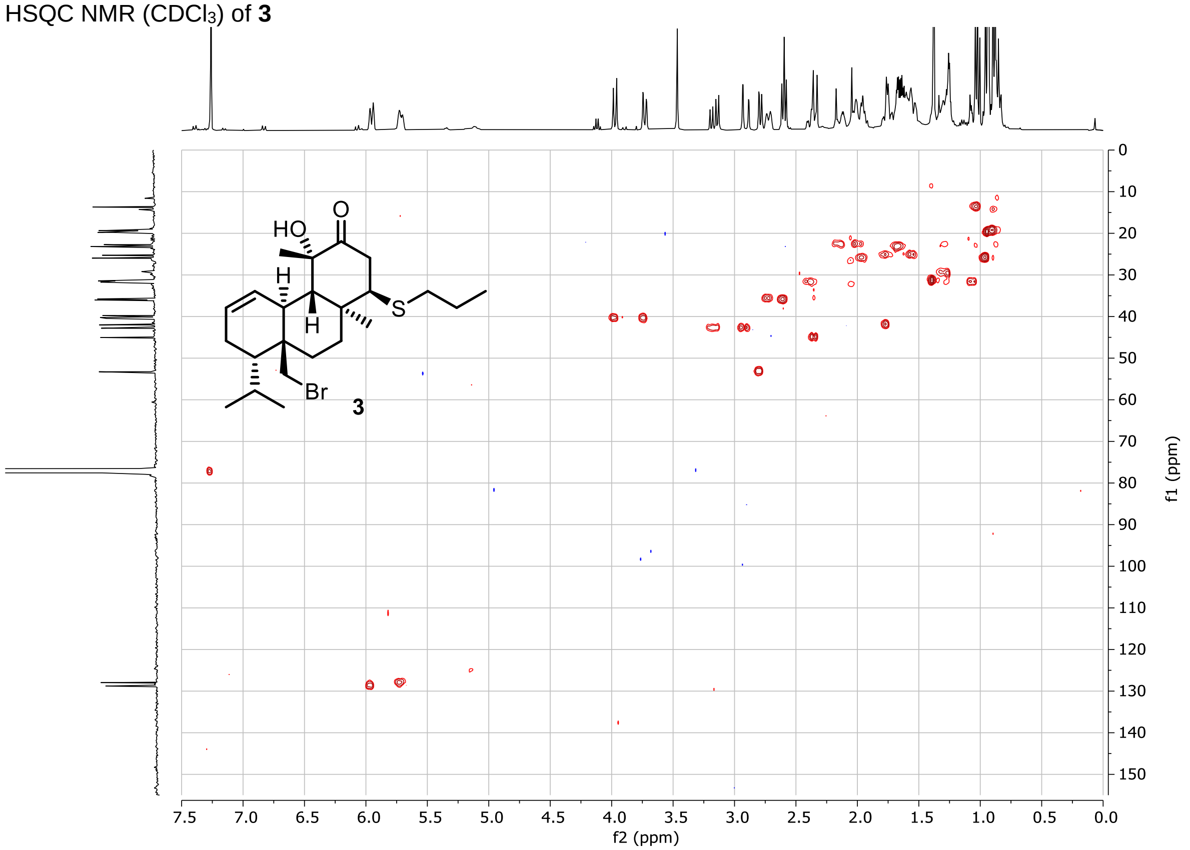

COSY NMR ($CDCl_3$) of **3**

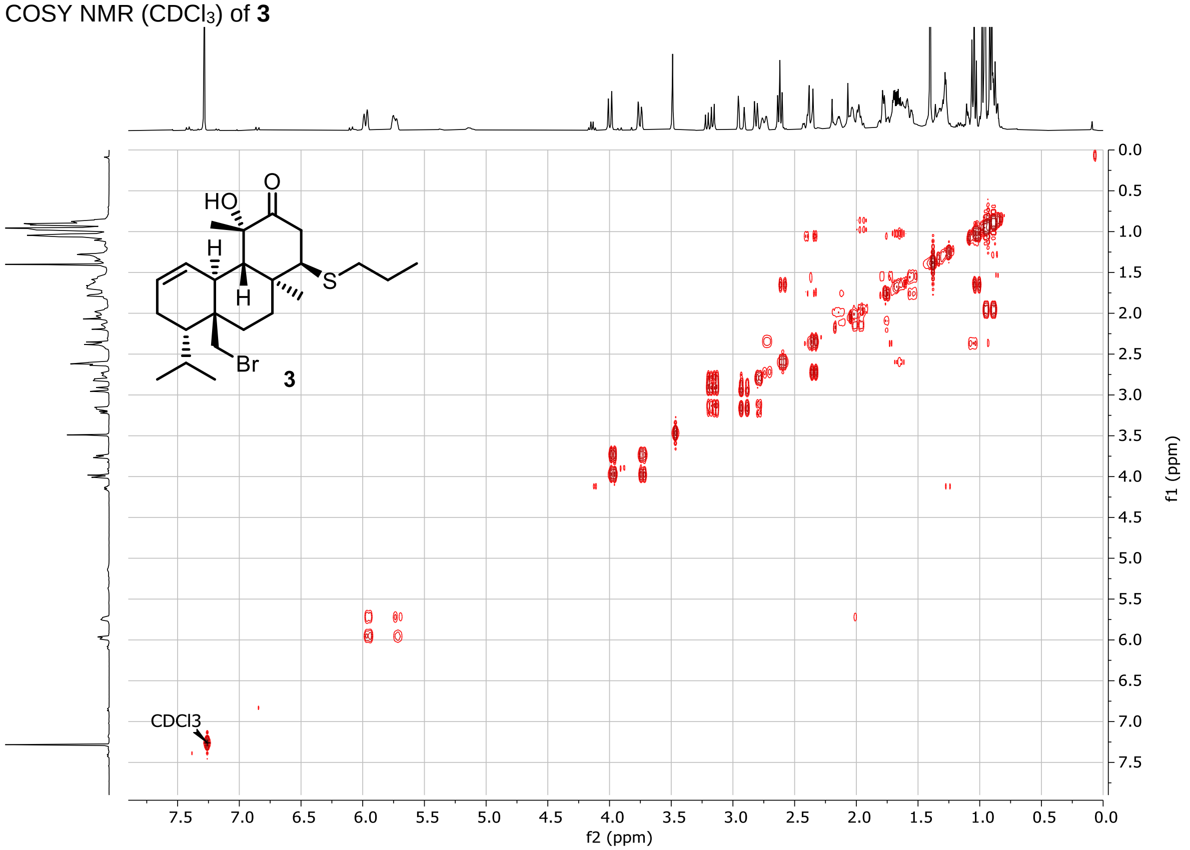

HRMS spectra of **3**

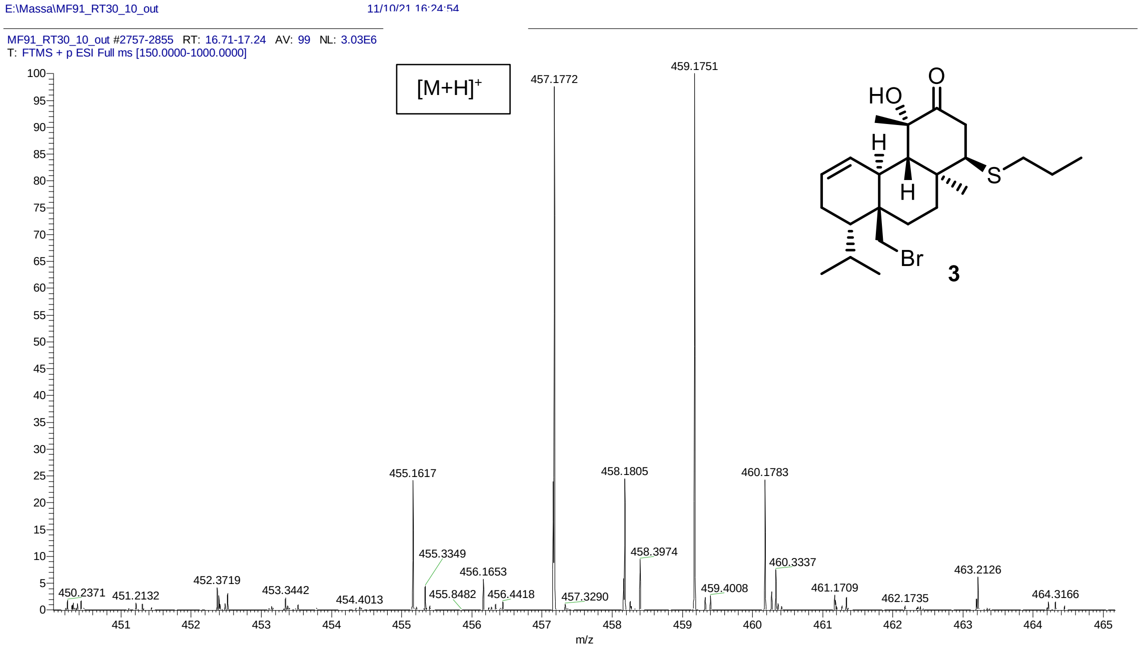

^{1}H NMR (300 MHz, $CDCl_3$) of **4**

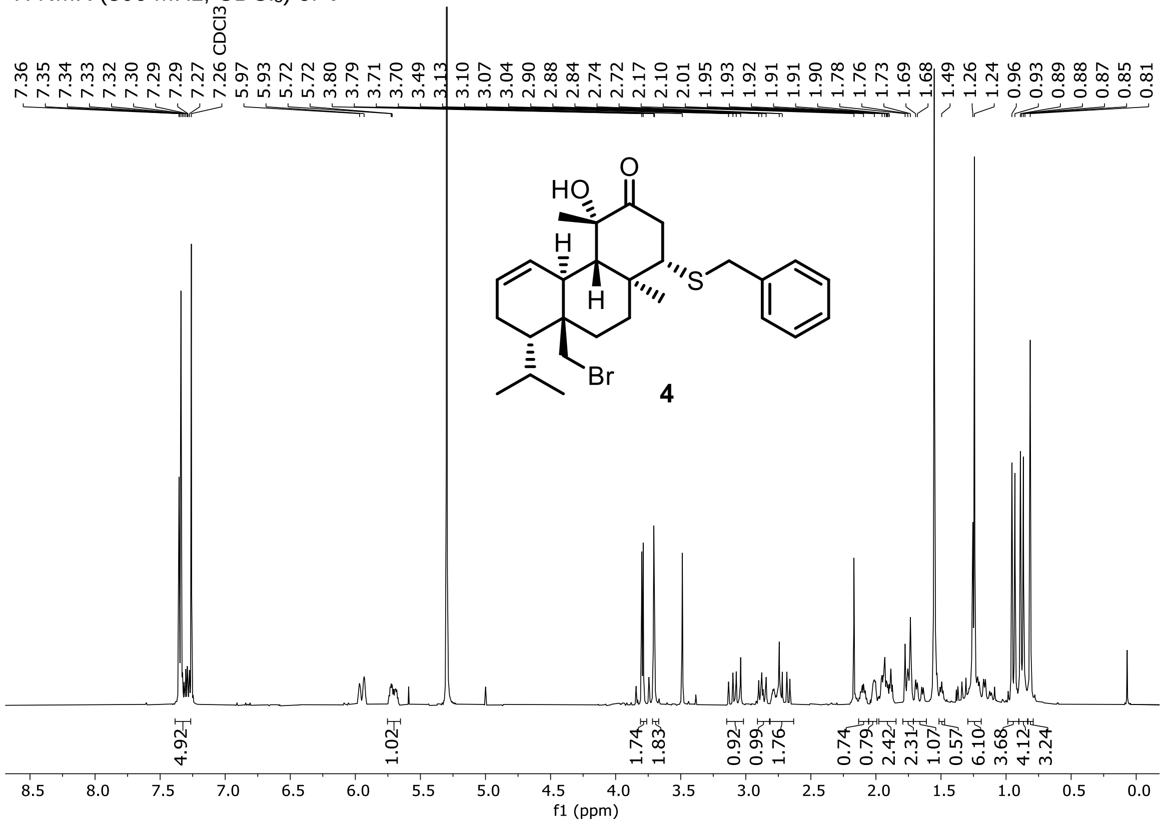

^{13}C NMR (101 MHz, $CDCl_3$) of **4**

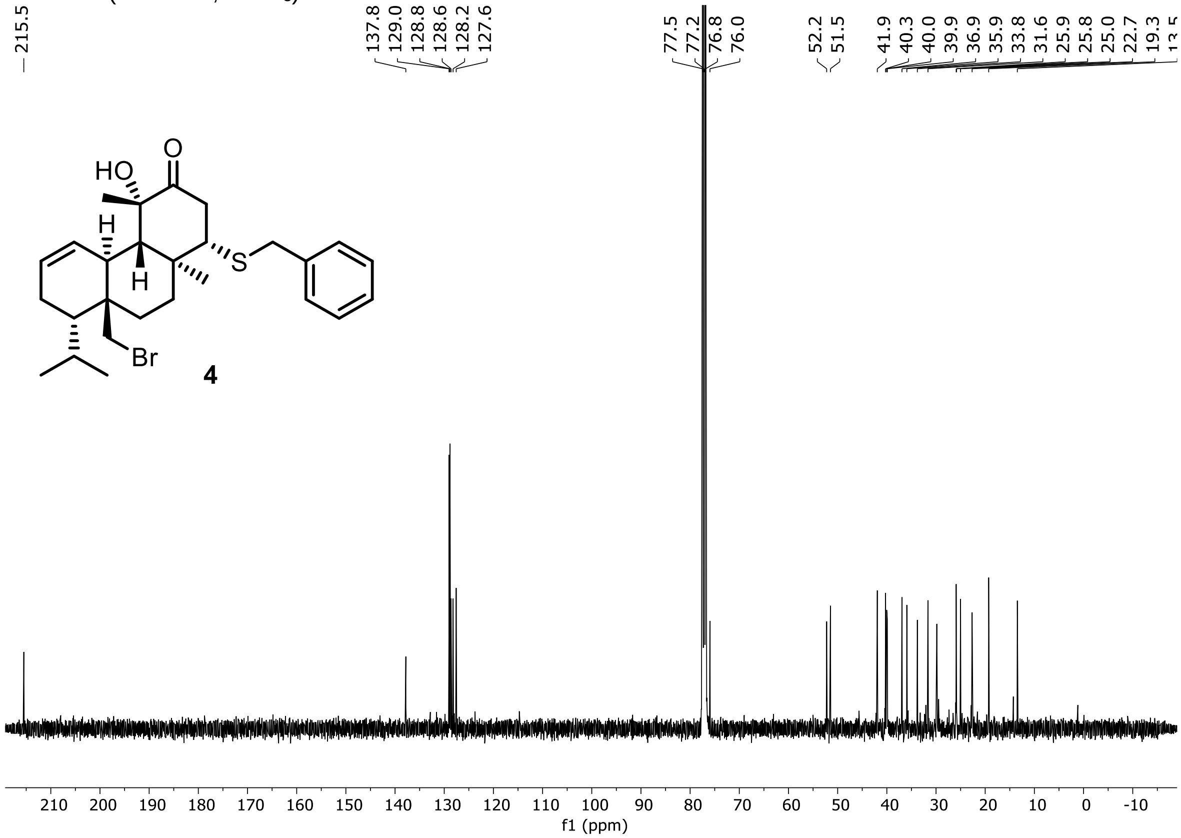

HSQC NMR ($CDCl_3$) of **4**

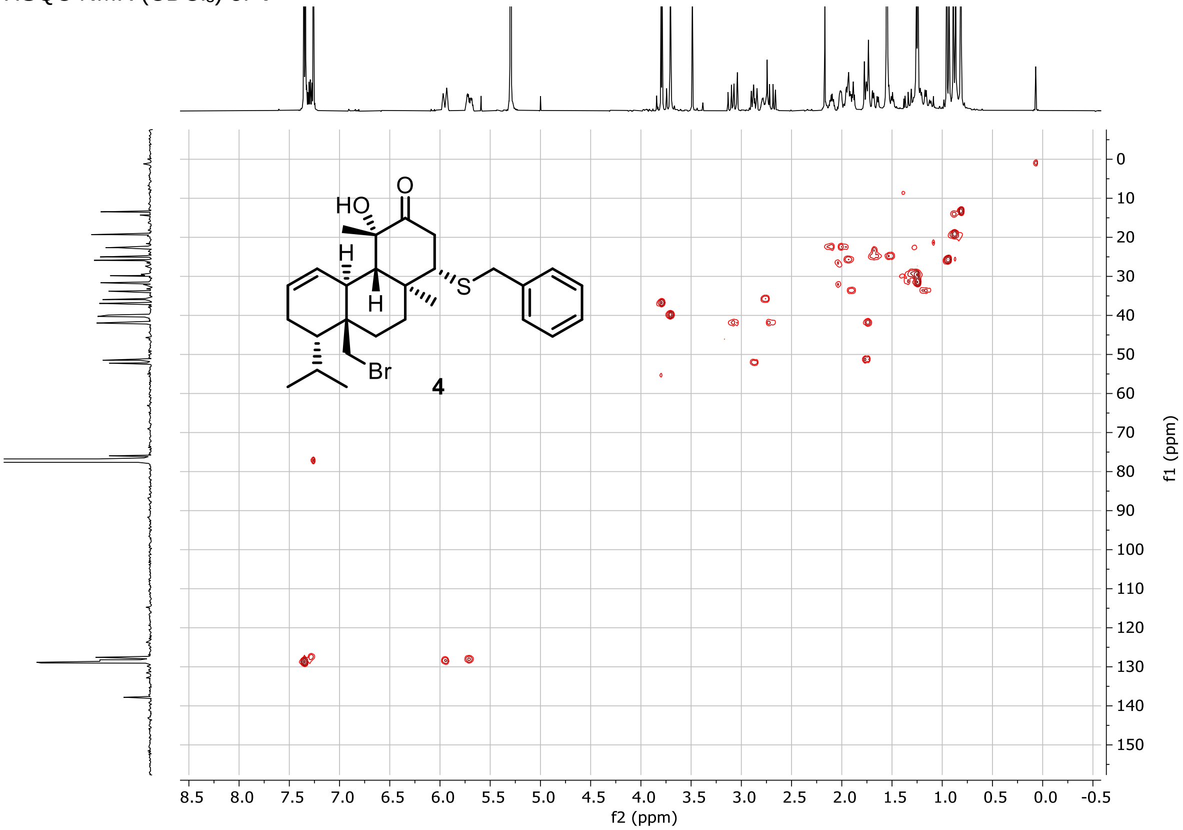

COSY NMR ($CDCl_3$) of **4**

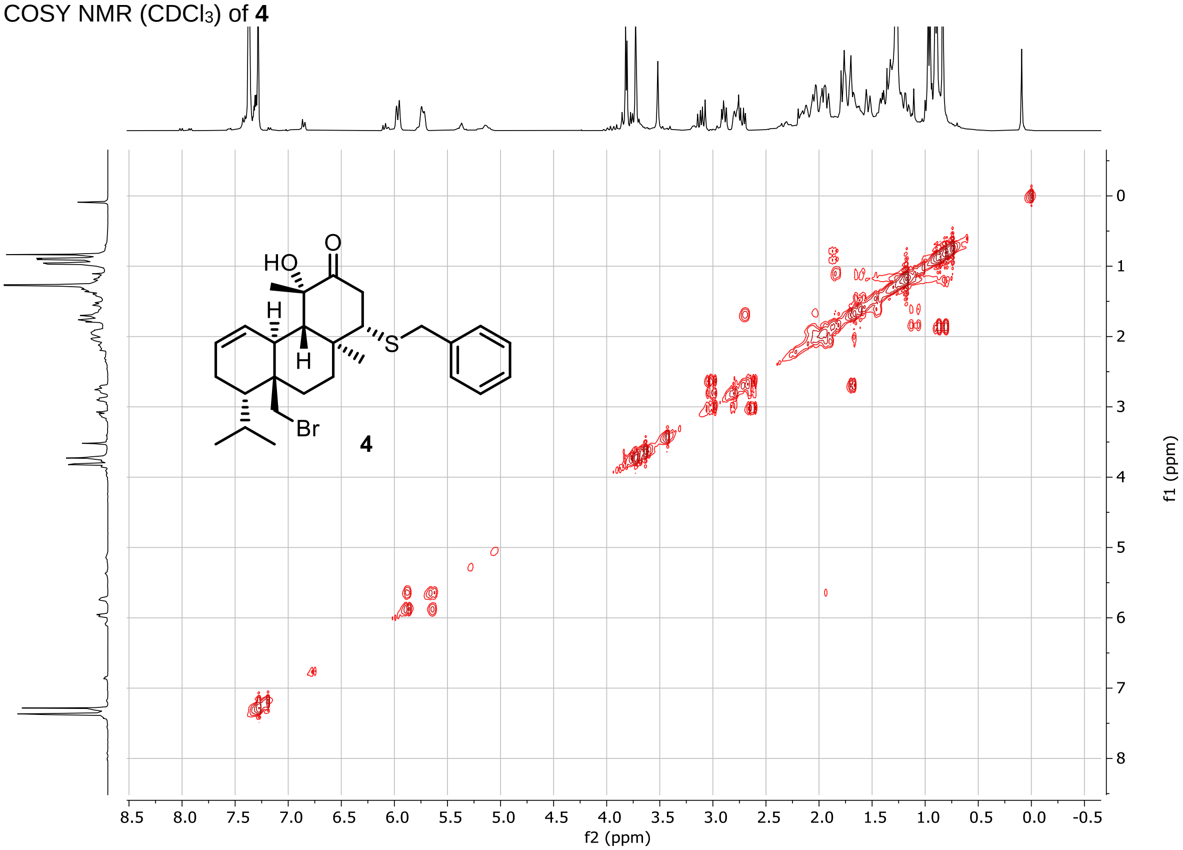

HRMS spectra of **4**

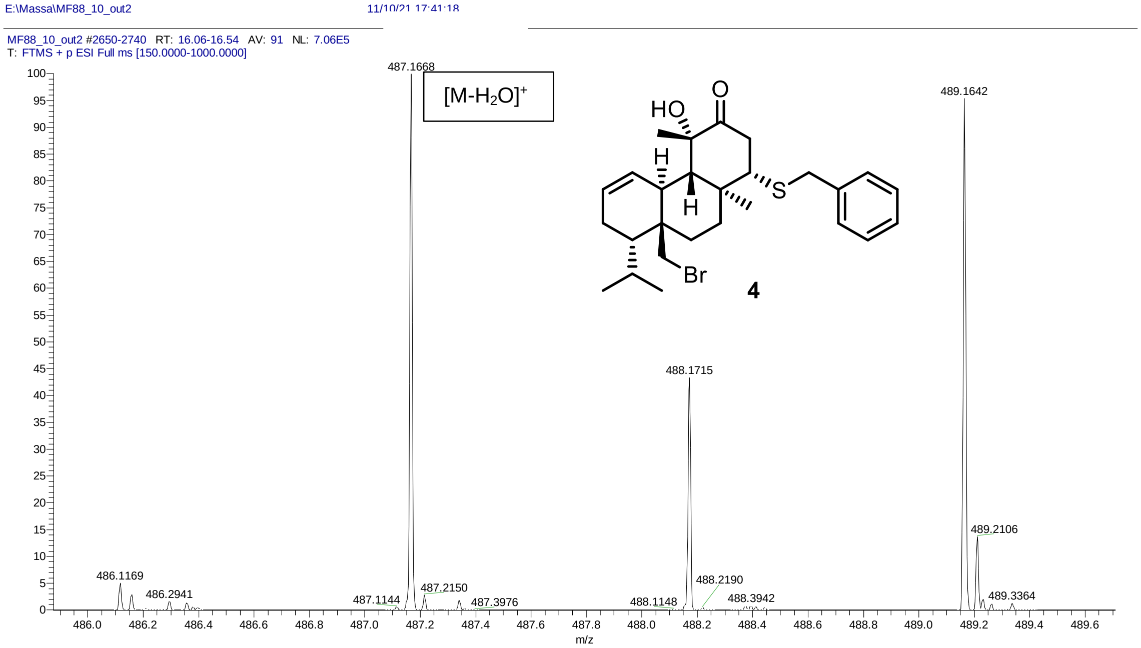

^{1}H NMR (400 MHz, $CDCl_3$) of **5**

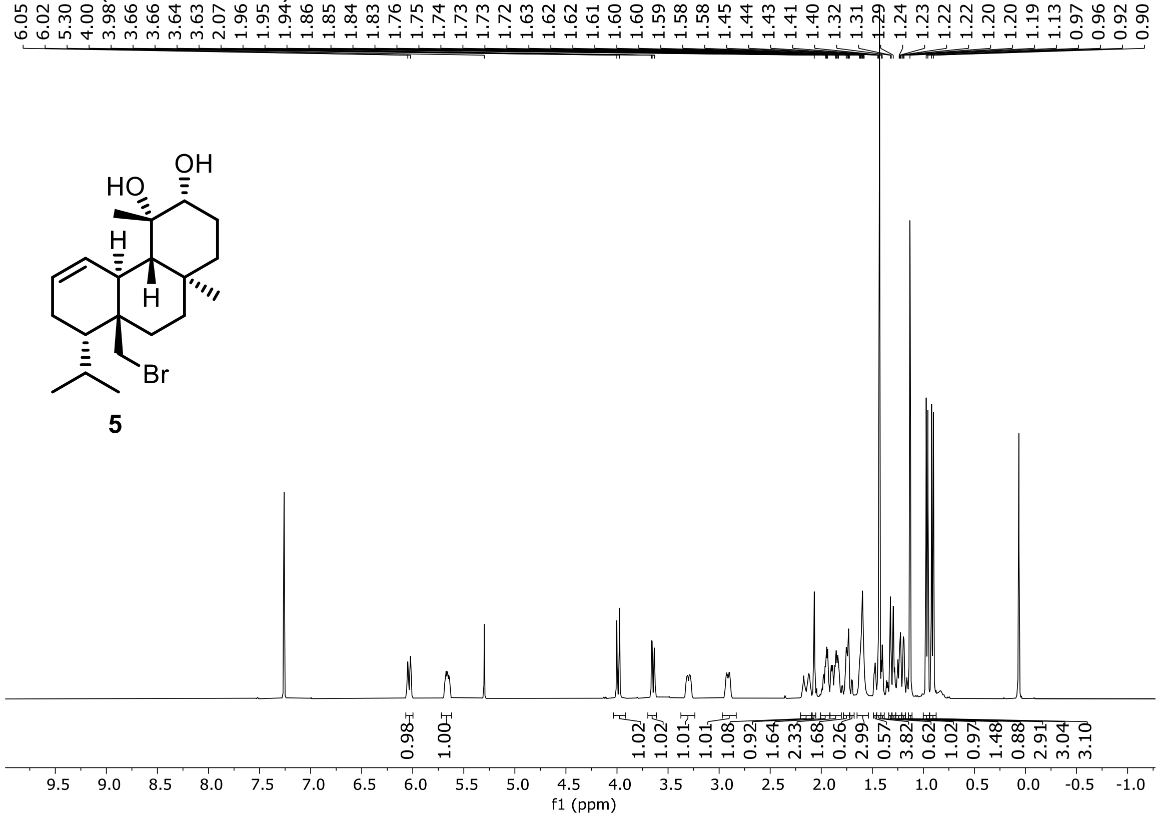

^{13}C NMR (101 MHz, $CDCl_3$) of **5**

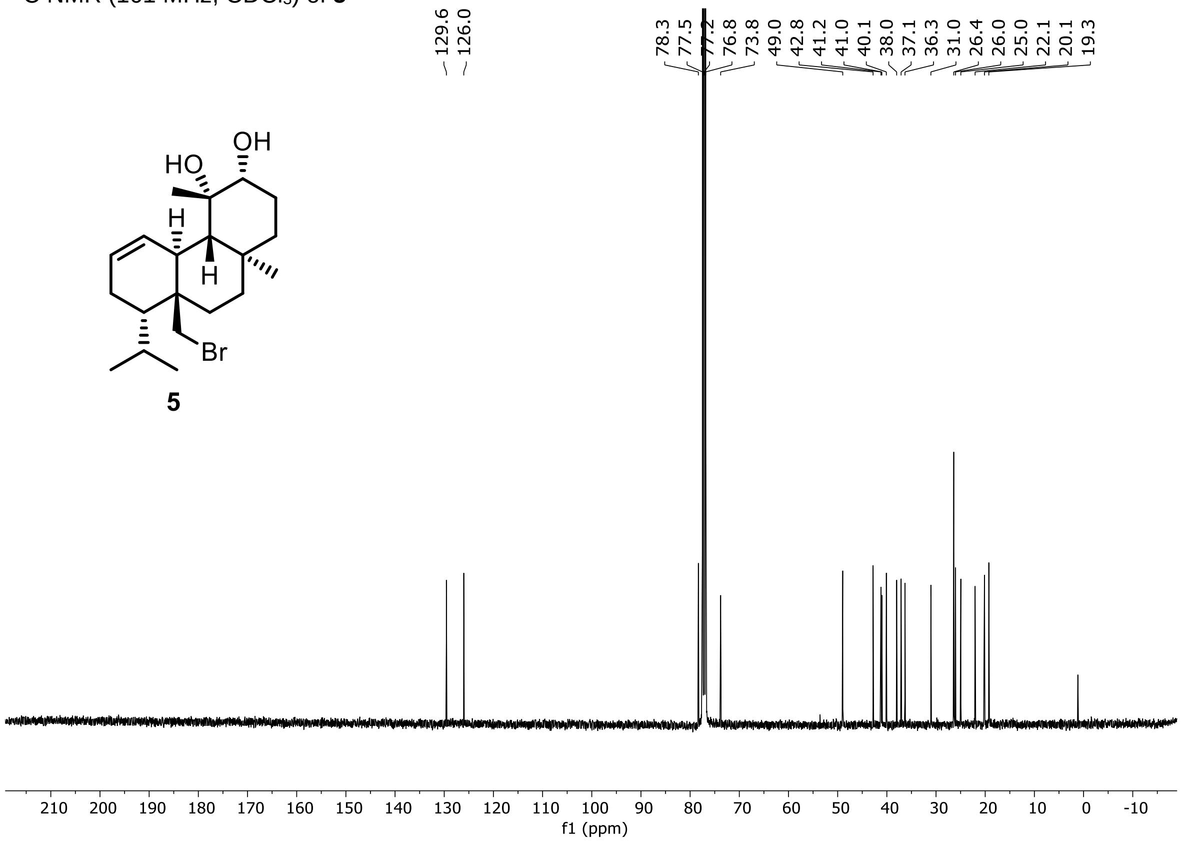

HSQC NMR ($CDCl_3$) of **5**

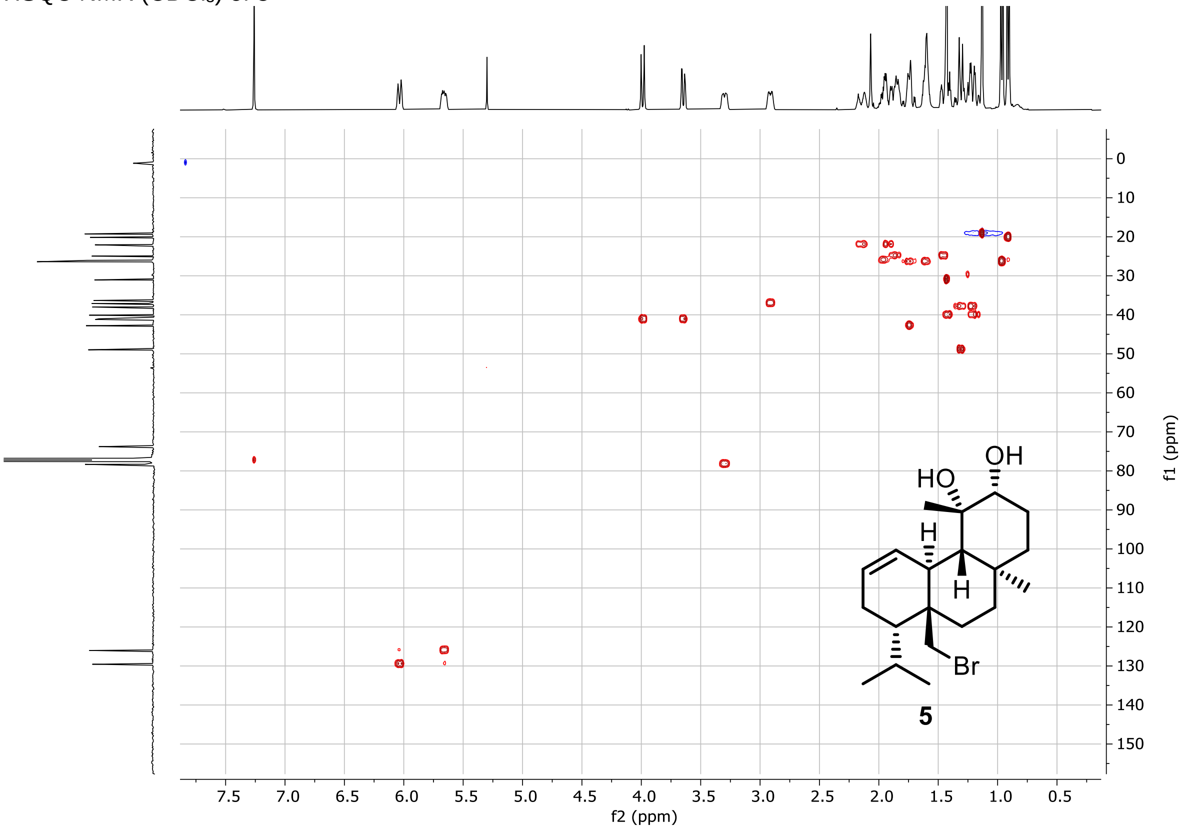

COSY NMR ($CDCl_3$) of **5**

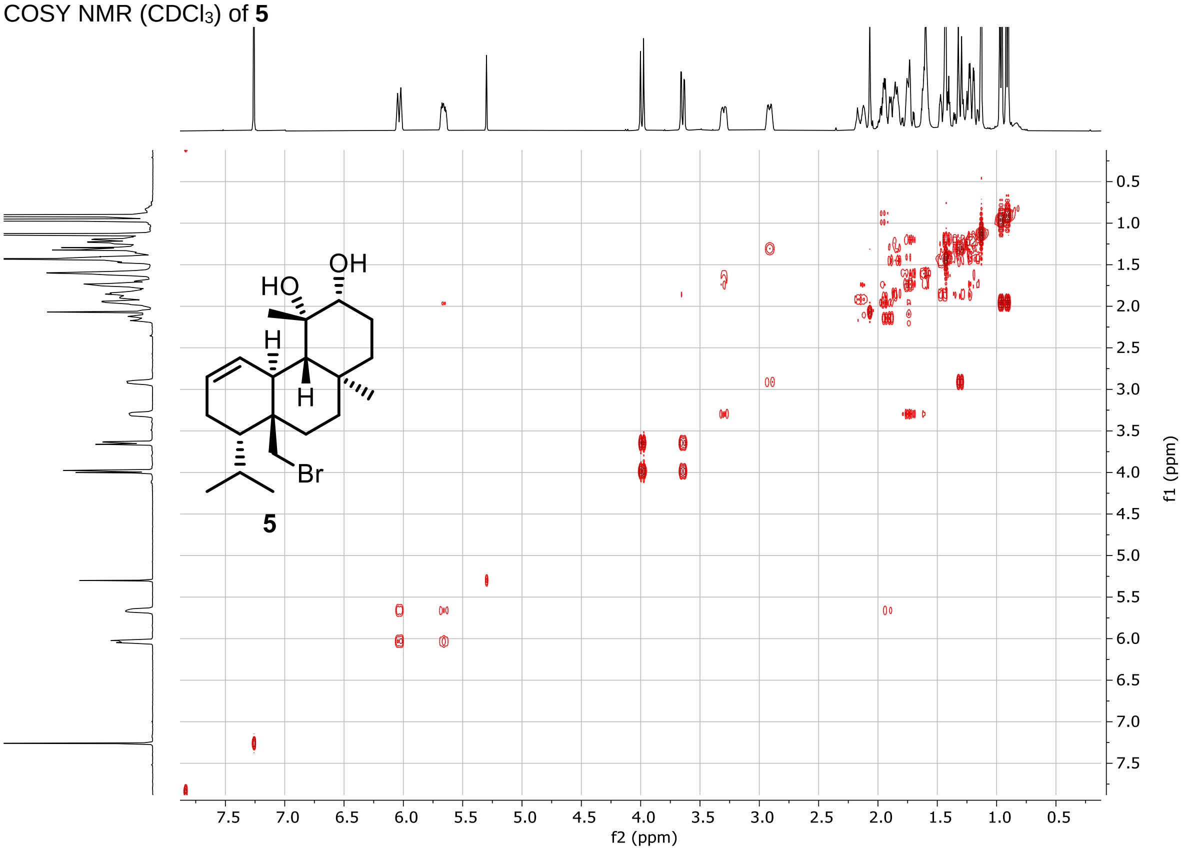

HRMS spectra of **5**

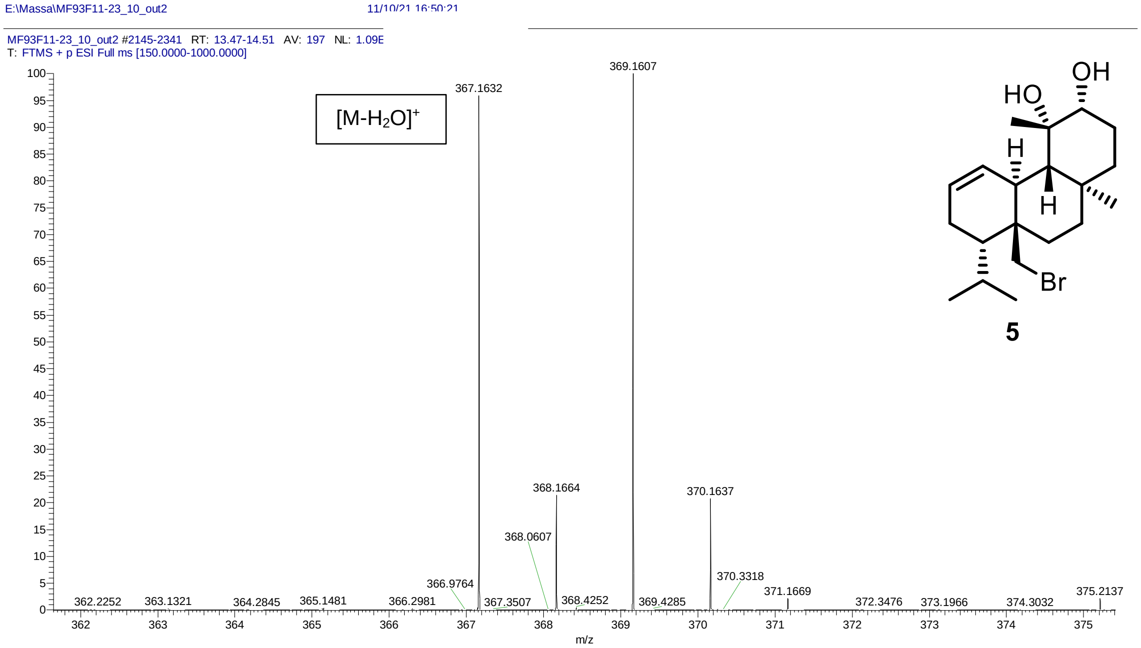

5

^{1}H NMR (300 MHz, $CDCl_3$) of **6**

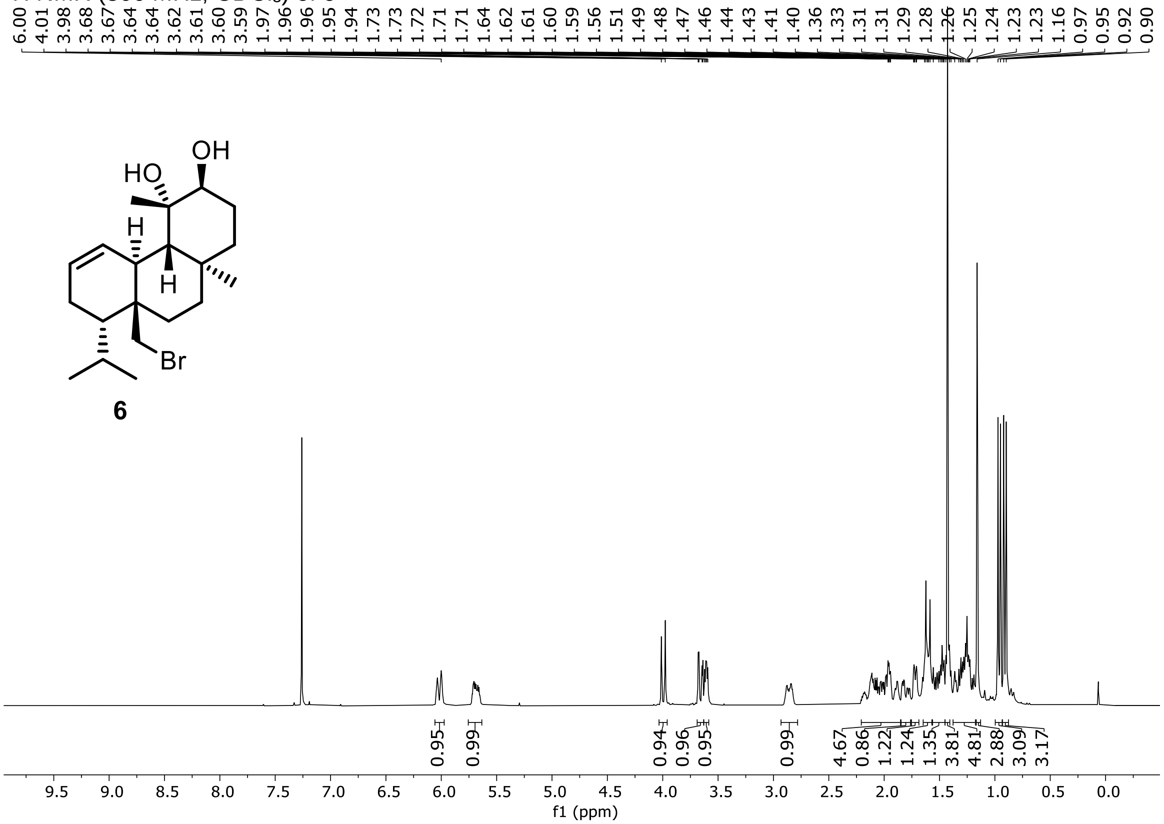

^{13}C NMR (75 MHz, $CDCl_3$) of **6**

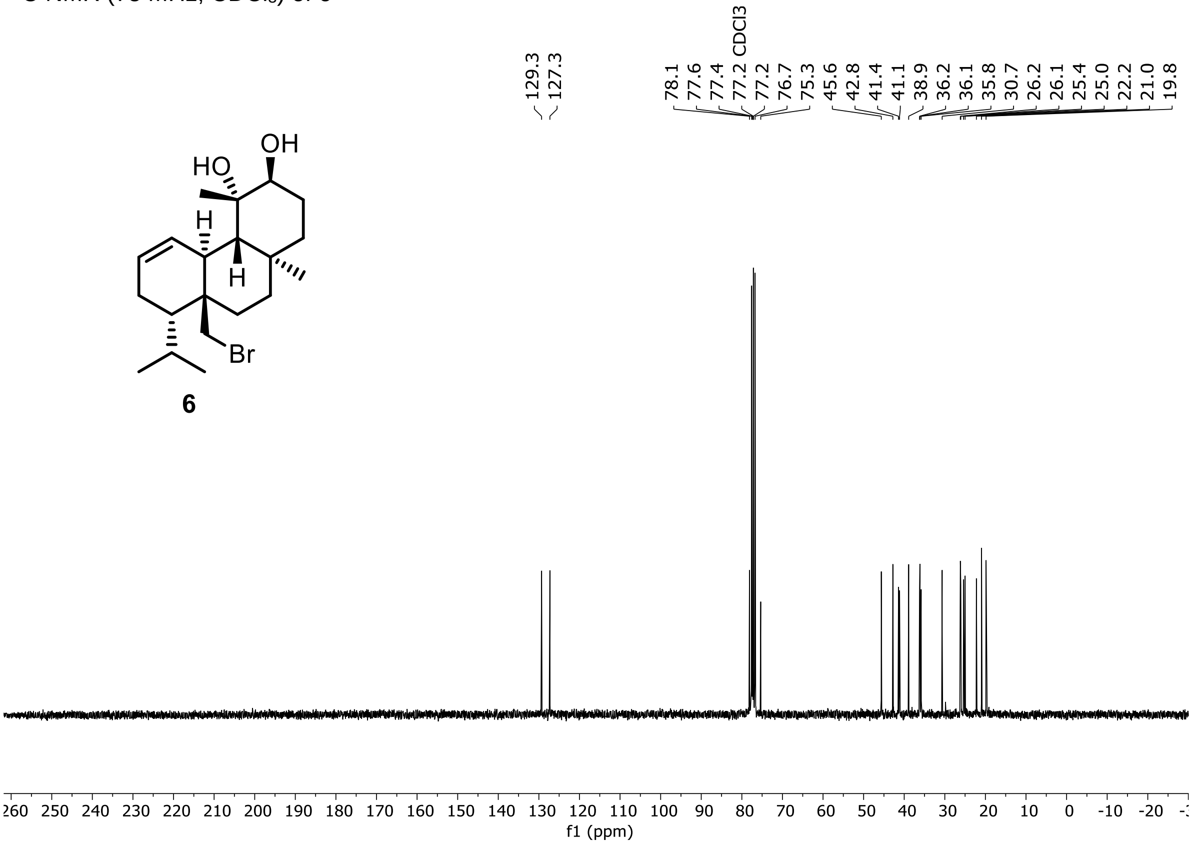

HSQC NMR ($CDCl_3$) of **6**

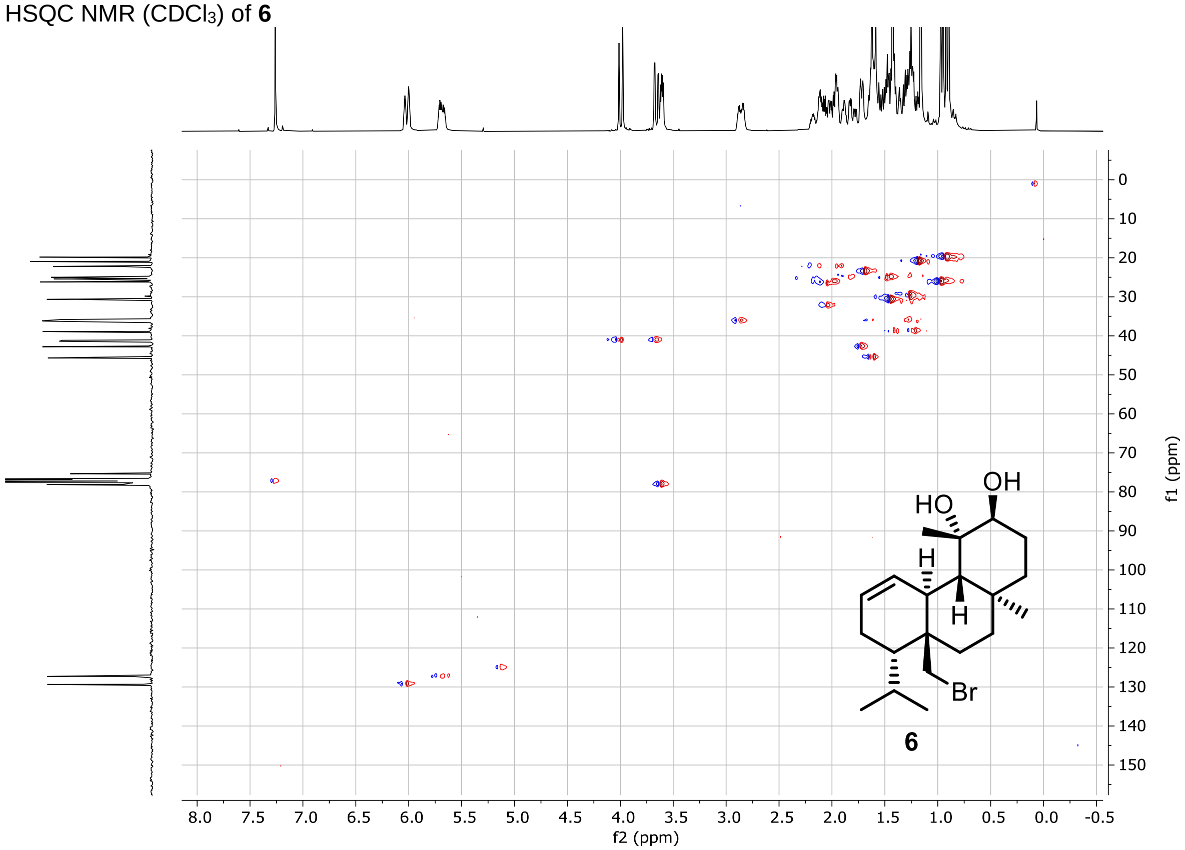

COSY NMR ($CDCl_3$) of **6**

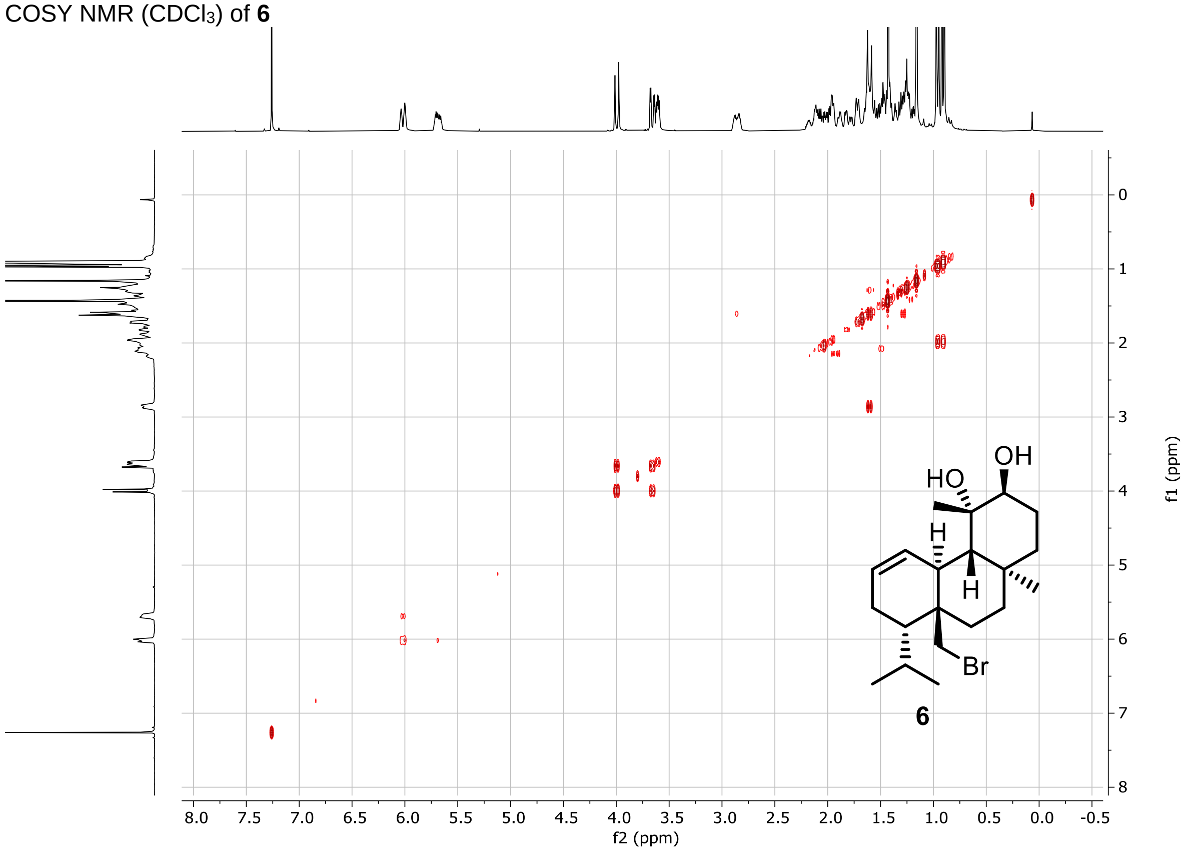

HRMS spectra of **6**

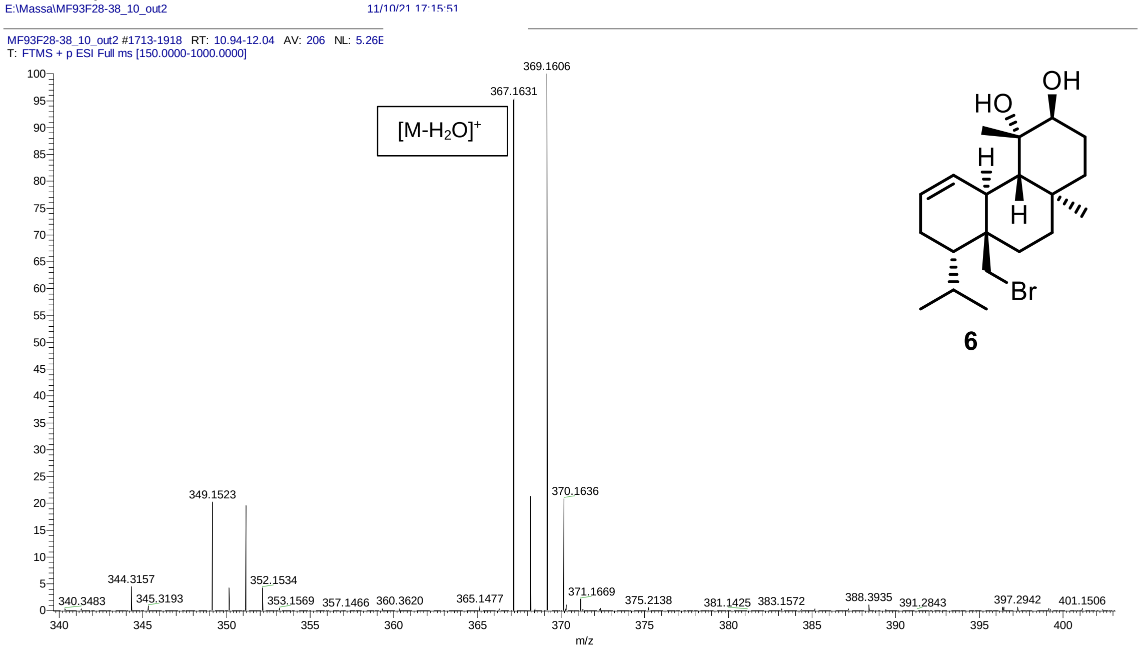

Supplement: Supplementary file 1 [file marinedrugs-22-00408-s001.zip › marinedrugs-3146078-supplementary.pdf]
